# Multi-label Contextual Operator Inference in Knowledge Acquisition for Building Generalization: A Multi-modal Learning Approach

**DOI:** 10.1007/s41651-026-00251-w

**Published:** 2026-04-01

**Authors:** Joris Senn, Zhiyong Zhou, Cheng Fu, Robert Weibel

**Affiliations:** 1 Department of Geography, University of Zurich, Zurich, Switzerland; 2 Department of Geography, University of Wisconsin–Madison, Madison, Wisconsin USA; 3 College of Information and Electrical Engineering, China Agricultural University, Beijing, China

**Keywords:** Building generalization, Generalization operators, Knowledge acquisition, Multiple operator selection, Multi-modal learning

## Abstract

In traditional map generalization, knowledge acquisition refers to formalizing individual generalization operators and chaining multiple operators into an overall process. It is an essential step towards automating map generalization. To date, machine learning, including advanced deep learning (DL), has been frequently applied to implement individual operators or end-to-end solutions, reducing or even bypassing the intensive effort required to elicit generalization knowledge from expert cartographers. For the example of the generalization of buildings in topographic maps, we focus on the inverse process, that is, how cartographic knowledge acquisition can be performed in DL-based map generalization, a task that has received little attention so far. In particular, we address *process recognition and modeling* and formulate the decision of whether or not multiple contextual generalization operators, including *enlargement*, *aggregation*, *typification*, and *displacement*, should be applied to a particular building as a *multi-label contextual operator inference* task. Instead of using either raster or vector maps to learn map generalization, we propose a *multi-modal* learning approach to explore the capacity of integrating both map data modalities. We evaluated the approach with national multi-scale map production data in Switzerland, for the transition from 1:25,000 to 1:50,000. The results show that trained DL models achieved F1 scores greater than 0.80 for operator inference except for *typification*, indicating the promising capacity of DL approaches to acquire procedural knowledge on building generalization. Furthermore, a closer evaluation by operator combinations demonstrates that the multi-modal approach particularly outperforms uni-modal learning in process recognition and modeling involving multiple operators.

## Introduction

Map generalization plays a fundamental role in managing a large amount of topographic map data and visualizing it at multiple scales (Duchêne et al. 2014). Since buildings are one of the key object classes represented on topographic maps, the generalization of buildings receives crucial attention within the overall map generalization process. It involves a series of operations, such as simplification, aggregation, and typification, on an individual or groups of buildings to maintain visually meaningful and legible maps when the scale is reduced from a large to a smaller scale (Regnauld and McMaster 2007). Due to the huge volume of data that exist for topographic maps, automated or semi-automated map generalization algorithms, including ones for building generalization, have been, and are being, developed based on knowledge bases (e.g., graphic examples) formalized by expert cartographers (Spiess et al. 2005), constituting one of the most conventional means of cartographic knowledge acquisition (Weibel 1995).

However, knowledge acquisition from cartographic experts is not only labor-intensive but also limited by the vagueness and difficulty of verbal or textual formalization (Weibel et al. 1995). This limitation is particularly magnified in formalizing the generalization process that involves multiple operators (Harrie and Weibel 2007). Machine learning (ML), including advanced deep learning (DL), has great potential to automate knowledge acquisition through the data-driven learning paradigm (Harrie et al. 2024; Kang et al. 2024; Weibel et al. 1995). To date, ML studies related to the explicit automation of knowledge acquisition mainly focus either of functions of structure and pattern recognition (Brassel and Weibel 1988; Sester 2000) preceding the actual generalization steps (Yan et al. 2019; Yu et al. 2022) or on implementations of individual generalization operators, such as selection (Xiao et al. 2024b) and simplification (Du et al. 2022; Yan and Yang 2024; Zhou et al. 2023). However, the generalization process modeling that involves decisions extending to multiple operators has received little attention. Although some studies have introduced end-to-end DL methods to generalize raster maps (Courtial et al. 2023; Feng et al. 2019; Fu et al. 2024; Zhou et al. 2024), they offer little insight into acquiring knowledge about the generalization process.

**Fig. 1:**
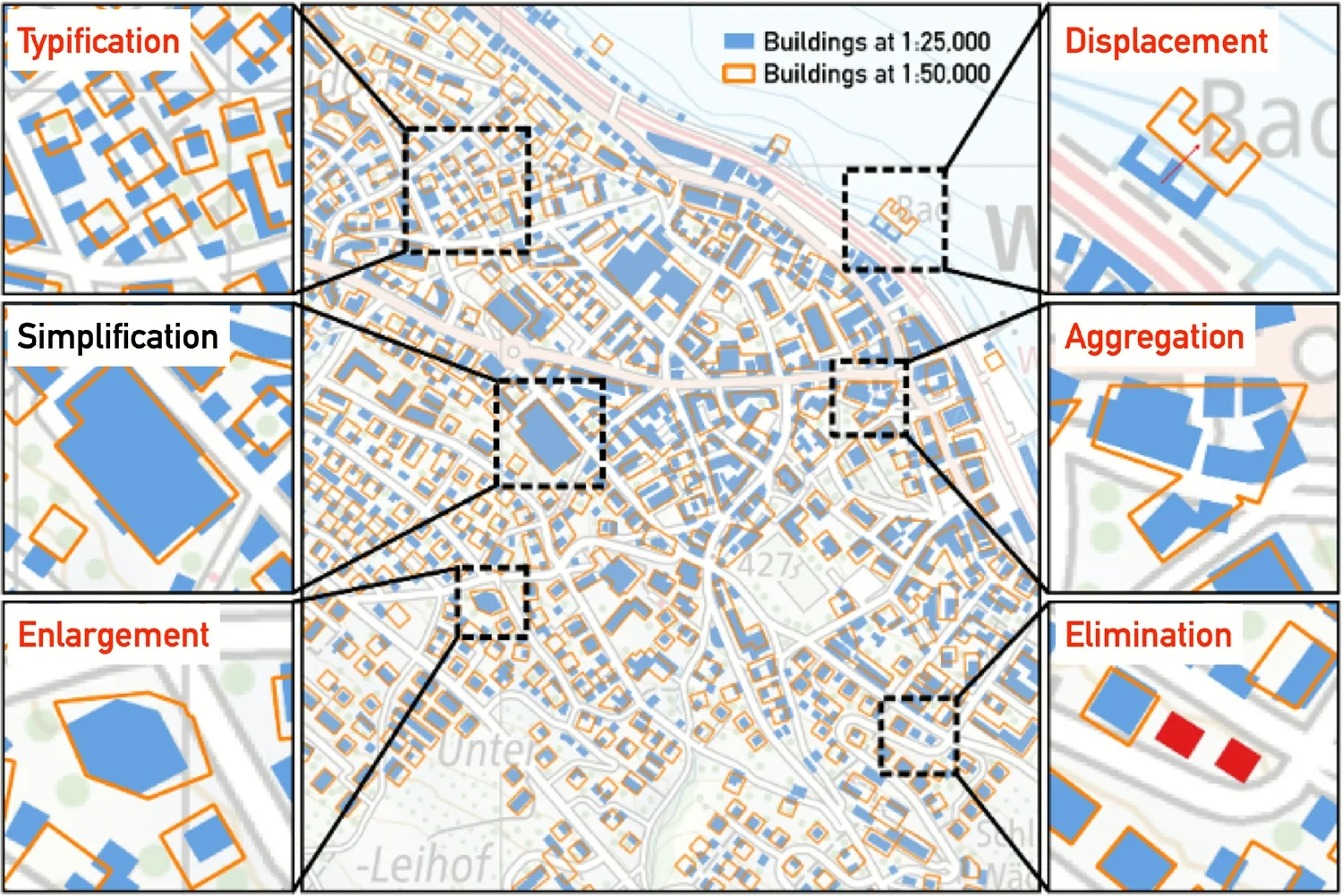
Graphical illustration of dominant building generalization operators (map data © swisstopo). The operators colored in red are regarded as contextual operators

To overcome the aforementioned limitation, we formalize a new deep learning task for knowledge acquisition in building generalization, termed *multi-label contextual operator inference*, akin to the *process recognition* and *process modeling* steps of the conceptual model of map generalization by Brassel and Weibel (1988). The task aims to infer whether or not particular building generalization operators should be applied to the generalization of a given building based on its surrounding context. As shown in Fig. 1, six operators, including *simplification*, *elimination*, *enlargement*, *aggregation*, *typification*, and *displacement*, are predominantly involved in building generalization. While *simplification* focuses on the reduction of geometric complexity of individual buildings and *elimination* typically takes place as a pre-processing step (Spiess et al. 2005, p. 42), the application of the remaining four operators—enlargement, aggregation, typification, and displacement—is commonly guided by contextual relationships of a building with other map features, and they often occur in combination with each other, necessitating the concurrent inference of multiple operators, rather than in isolation. Hence, we deal with these four contextual operators in this article.

In deep learning for map generalization, raster and vector maps have different advantages as input. The former offers a unified way to encode map objects (Feng et al. 2019; Yu and Chen 2022), while the latter is more concise in representing the contextual relationships among map objects and thus more efficient in learning generalization (Cui et al. 2025). Therefore, an intuitive hypothesis is that the integration of these two modalities of map data can further benefit the *multi-label contextual operator inference* task in knowledge acquisition for building generalization as defined above. The idea of such an integration of more than one data modality for the downstream learning task is typically called *multi-modal learning* (Baltrušaitis et al. 2018). It is not new to many subdomains of geospatial AI, such as remote sensing and urban computing (Mai et al. 2025), but its capacity for computational cartography, including map generalization, has rarely been explored.

In this article, we propose a multi-modal learning model for *multi-label operator inference* in knowledge acquisition for building generalization. Given a map depicting street blocks containing buildings and streets that need to be generalized when producing a map of a smaller scale, our approach simultaneously adopts a three-channel raster data model and a graph data model to represent the street block map and inputs them to extract raster and vector map features, respectively. A four-head classification module is designed to infer the four operators for building generalization mentioned above. The contributions of our work are threefold:

- Conceptually, rather than focusing on operator implementation or end-to-end solutions by DL models, like most existing studies, we address the inverse problem, how cartographic knowledge acquisition can be performed in DL-based map generalization. For the first time, we formulate the operator decision problem in the process recognition and process modeling of building generalization as a *multi-label operator inference* task, which is learnable by deep learning models.
- The proposed multi-operator, multi-modal learning model was evaluated with the national multi-scale map production data by the Swiss Federal Office of Topography (swisstopo), DKM25 (digital cartographic model at 1:25,000) and DKM50 (at 1:50,000).^1^ As a result, trained DL models achieved F1 scores all greater than 0.8 for operator inference except for *typification*, showing the promising capability of DL in acquiring procedural knowledge on building generalization.
- A closer evaluation by the operator combinations demonstrates that the multi-modal model outperforms uni-modal models (i.e., raster or vector), particularly in the inference of operator combinations. Despite there being room for improvement, this indicates the greater potential of multi-modal learning in acquiring process modeling knowledge for building generalization compared to uni-modal learning.

## Related Work

### Process Modeling of Map Generalization

Map generalization process modeling refers to the chaining or coordination of multiple elementary generalization operators (e.g., simplification and aggregation) with certain control parameters (Harrie and Weibel 2007). In traditional map generalization, the overall generalization process is seen holistically by simultaneously considering multiple map objects and applying multiple generalization operators to resolve potential conflicts of map legibility (Brassel and Weibel 1988). This requires a higher level of map knowledge acquisition than defining individual operators (Müller 1991; Sarjakoski 2007). Traditional process modeling is explicitly defined by expert cartographers, with support from computer systems (Harrie and Weibel 2007). It mainly follows three paradigms: condition-action modeling, human interaction modeling, and constraint-based modeling.

To date, the constraint-based modeling paradigm has resulted in the most satisfying results with the least human intervention needed for map generalization. In such a paradigm, the map specifications can be formalized in the form of various constraints that must be met to the best possible extent during an iterative generalization process (Beard 1991). This paradigm allows for the evaluation of different strategies that support backtracking should the result not be satisfactory (Harrie and Weibel 2007; Mackaness 1995). The best-performing methodology for constraint-based map generalization is the agent-based approach that models map objects as a set of communicating and interacting agents whose target is to achieve an optimal generalization state for themselves and other map objects (Barrault et al. 2001; Duchêne et al. 2018; Ruas and Duchêne 2007; Sarjakoski 2007). In production environments, often the technically demanding agent-based approach has been replaced by a workflow approach (Stoter et al. 2014). Moreover, other optimization techniques such as genetic algorithms (Wilson et al. 2003), least-squares adjustment (Sester 2005), elastic beams (Bader et al. 2005), and simulated annealing (Ware et al. 2003) have also been applied to constraint-based map generalization.

### Machine Learning for Building Generalization

Beyond rules or constraints that are clearly defined by expert cartographers, a lot of generalization knowledge, including process recognition and modeling, is implicit and difficult to describe and parameterize, even though expert cartographers can generalize example cases. This limitation has been called the cartographic knowledge acquisition bottleneck (Sester 2000; Weibel et al. 1995). Thus, machine learning (ML) techniques were applied to existing generalized maps to learn the implicitly encoded knowledge infused by expert cartographers during manual map generalization (Sester 2020). It is essentially reverse engineering the generalization process. Initial generalization approaches based on ML were concerned with the structure characterization of individual map objects (Steiniger et al. 2008), pattern or relation recognition from multiple map objects (Sester 2000; Steiniger et al. 2008), parameter estimations for generalization operators (Lagrange et al. 2000; Plazanet et al. 1998; Weibel et al. 1995), and implementations of individual generalization operators (Cheng et al. 2015, 2013).

The advancement of deep learning has offered a new paradigm for automated map generalization (Touya et al. 2019), as it has a superior capability over traditional machine learning in processing raw map data and requires less feature engineering to achieve satisfactory performance. The existing DL studies for map generalization are mainly aimed at the generalization of topographic features, including buildings and roads. Particularly, these studies predominantly focus on end-to-end generalization for raster building maps (Courtial et al. 2023; Feng et al. 2019; Fu et al. 2024; Zhou et al. 2024) without considering intermediate procedural knowledge, with the exception of Fu et al. (2024), who made some first steps in this area. For the generalization of vector mode maps, work has focused on implementations of individual building generalization operators, such as aggregation (Yan et al. 2022) and simplification (Du et al. 2022; Jiang et al. 2023; Zhou et al. 2023). Moreover, the multiple modalities of maps have commonly been ignored in the research on automated map generalization (Janowicz et al. 2025), although several attempts have fused multiple modalities of other geospatial data, such as remote sensing images and language (Liu et al. 2024; Vivanco Cepeda et al. 2023), and vector geometry and textual descriptions of points of interest (Balsebre et al. 2024).

### Research Gaps

In summary, process modeling of map generalization today typically utilizes constraint-based approaches, combined with optimization techniques, or workflow-based approaches, and involves a set of constraints with certain parameters. Deep learning is promising for the acquisition of map generalization knowledge, including building generalization. However, important research gaps remains.

First, apart from rather simple constraints such as those governing map legibility, explicitly modeling constraints is difficult. And modeling the procedural knowledge, that is, the generalization operators, algorithms, and parameter settings resulting from violations of constraints, is even harder. Furthermore, constraints are fixed and thus lack the flexibility to adapt to the varying scenarios of map generalization.

Second, prior studies on DL-based map generalization focused mainly on end-to-end solutions, but rarely on the entire process of building generalization, including multiple operators. This leads to a lack of operator-specific guidance for learning generalization, in particular with respect to procedural knowledge acquisition.

And third, although maps can be represented in multiple data modalities (i.e., raster or vector), existing DL approaches solely learn map generalization in a uni-modal form. It remains unknown to what extent the multi-modal learning paradigm can benefit building generalization, specifically the multi-label operator inference task formulated in this study.

## Methodology

### Concepts and Terminology

As illustrated in Fig. 1, the operators commonly applied in building generalization mainly include *simplification*, *elimination*, *enlargement*, *aggregation*, *typification*, and *displacement*. In this article, we focus mainly on the latter four so-called context-dependent operators whose application is guided by the surrounding cartographic features of a given building. The *simplification* operator is excluded from our study because its application modifies the geometries of individual buildings and is therefore not significantly affected by the presence of contextual buildings. We also did not include the *elimination* operator because it typically occurs before other operators in a sequential generalization process (Spiess et al. 2005, p. 42) and thus is mutually exclusive with the other operators, although it is context-dependent. Another reason for not considering *elimination* is that this study focuses on the medium-scale transition from 1:25,000 (source scale) to 1:50,000 (target scale), where *elimination* does not dominate the mix of generalization operators (Cecconi et al. 2002).

The four contextual operators considered here (Fig. 1) can be defined as follows (Regnauld and McMaster 2007; Stanislawski et al. 2014):

#### Definition 1

(Enlargement) The area of a building is increased to compensate for scale reduction, while maintaining shape clarity. In view of the formal problem statement below, it is labeled $l_{e} \in \{0, 1\}$ for a given building, where 0 stands for not being enlarged, while 1 stands for being enlarged.

#### Definition 2

(Aggregation) Two or more buildings of the source-scale map are represented as a new placeholder building in the target-scale map with a similar shape, orientation and aggregate size as the source group of buildings. It is labeled $l_{a} \in \{0, 1\}$ for a given building, where 0 stands for not being aggregated, while 1 stands for being aggregated.

#### Definition 3

(Typification) A group of buildings of the source-scale map are replaced by fewer buildings in the target-scale map, involving significant re-arrangement and re-symbolization, while attempting to maintain the patterns (e.g., geometric alignments) represented by the source group of buildings. Typification can also be seen as a combination of all other operators. It is labeled $l_{t} \in \{0, 1\}$ for a given building, where 0 stands for not being typified, while 1 stands for being typified.

#### Definition 4

(Displacement) The location of a building is adjusted (moved) to avoid overlap and congestion conflicts with other map features, while retaining the shape of the building and maintaining topological integrity with surrounding features. It is labeled $l_{d} \in \{0, 1\}$ for a given building, where 0 stands for not being displaced, while 1 stands for being displaced.

**Fig. 2:**
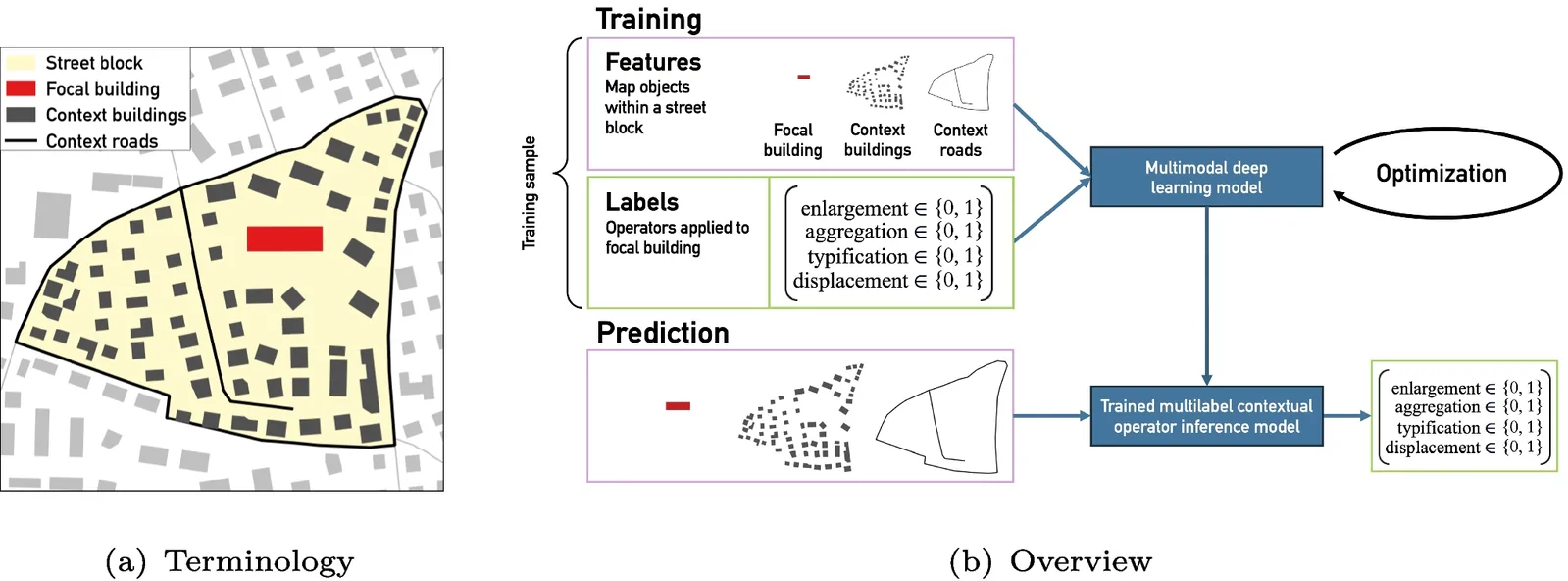
Conceptualization of the operator classification approach (map data © swisstopo)

Additionally, we need to define several concepts that are used in the problem formulation below as well as in the subsequent description of the proposed methodology (for an illustration, see Fig. 2a):

#### Definition 5

(Street block) An area enclosed by the road network on all sides, denoted by *st*.*blk*. The roads bounding the *street block*, including any dead-end roads lying within the block, are termed *context roads*, denoted by *ct*.*rds*.

#### Definition 6

(Focal building) Given a *street block* of buildings, a building that is aimed to be generalized is termed the *focal building*, denoted by *fc*.*bldg*.

#### Definition 7

(Context buildings) Given a *street block* of buildings, the remaining ones, except the *focal building*, are termed the *context buildings* to characterize the surroundings for generalization of the *focal building*n, denoted by *ct*.*bldgs*.

### Problem Formulation and Method Overview

As illustrated in Fig. 2, given a map of a *street block* with one or more data modalities, $M_{st.blk} = \{m_{1}, m_{2},... m_{i}\}$, where *i* refers to the *i*th type of map data modality and $m_{i} = \{fc.bldg_{i}, ct.bldgs_{i}, ct.rds_{i}\}$, the *contextual operator inference* for a focal building *fc*.*bldg* in the present study returns a four-dimension label vector $L_{fc.bldg} = <l_{a}, l_{t}, l_{d}, l_{e}>$ to decide if the *fc*.*bldg* should be *aggregated* with its context buildings, *typified*, *displaced*, and/or *enlarged*. Hence, the essential goal of the proposed method is to learn a function $f: M_{st.blk} \rightarrow L_{fc.bldg}$.

**Fig. 3:**
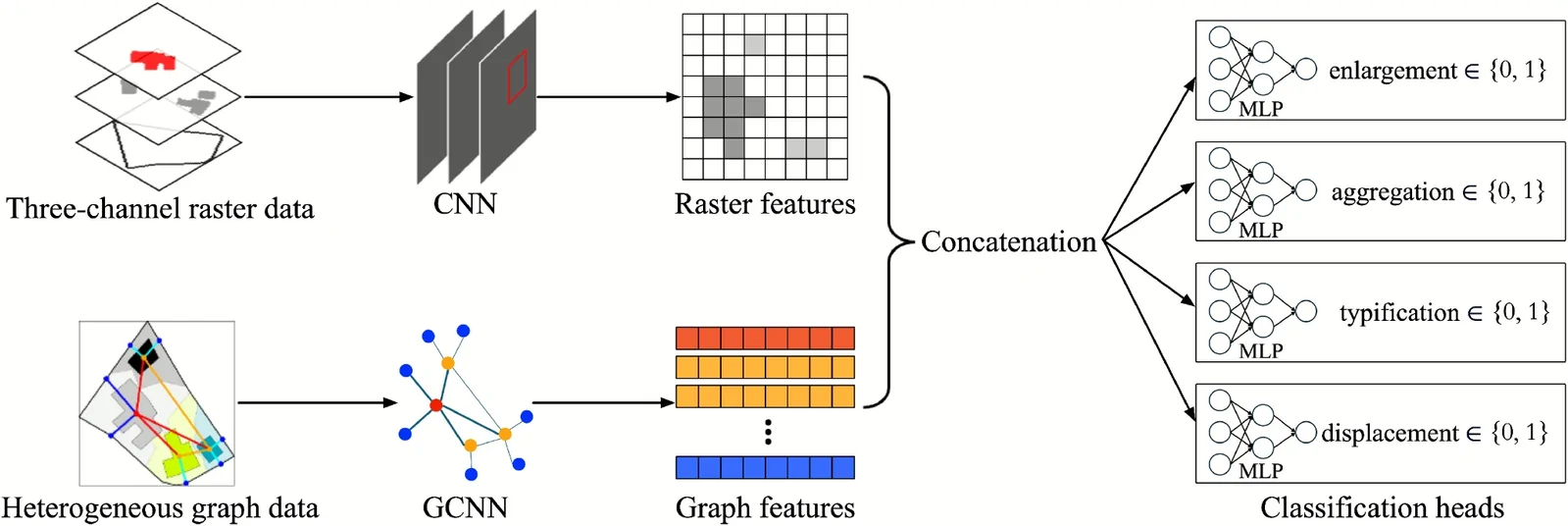
The architecture of the proposed methodology

Inspired by the various advantages of raster and vector data formats in representing map information, we introduce the multi-modal deep learning paradigm to extract informative features from different map data and propose a multi-head classifier for contextual operator inference in building generalization. As illustrated in Fig. 3, a three-channel raster data model and a heterogeneous graph data model (built on top of a vector map) are proposed to represent the information of individual map objects and spatial relationships among features in a street block map, respectively. Subsequently, they are input to a convolutional neural network (CNN) and a graph convolutional neural network (GCNN) to extract informative raster features and graph features of the street block map, followed by a concatenation of the features of the two data modalities. Finally, the concatenated features are passed to four classification heads, each of which infers the presence of a building generalization operator with a multi-layer perceptron (MLP) module. The following sections describe the main elements of the proposed methodology.

### Data Models for Multi-modal Maps

Two data modalities of maps are considered for the contextual building generalization operator inference: raster map and vector map. We developed a three-channel raster data model to encode the raster map information and a heterogeneous graph data model to represent the object information given in the vector map.

#### Three-channel Raster Data Model

**Fig. 4:**
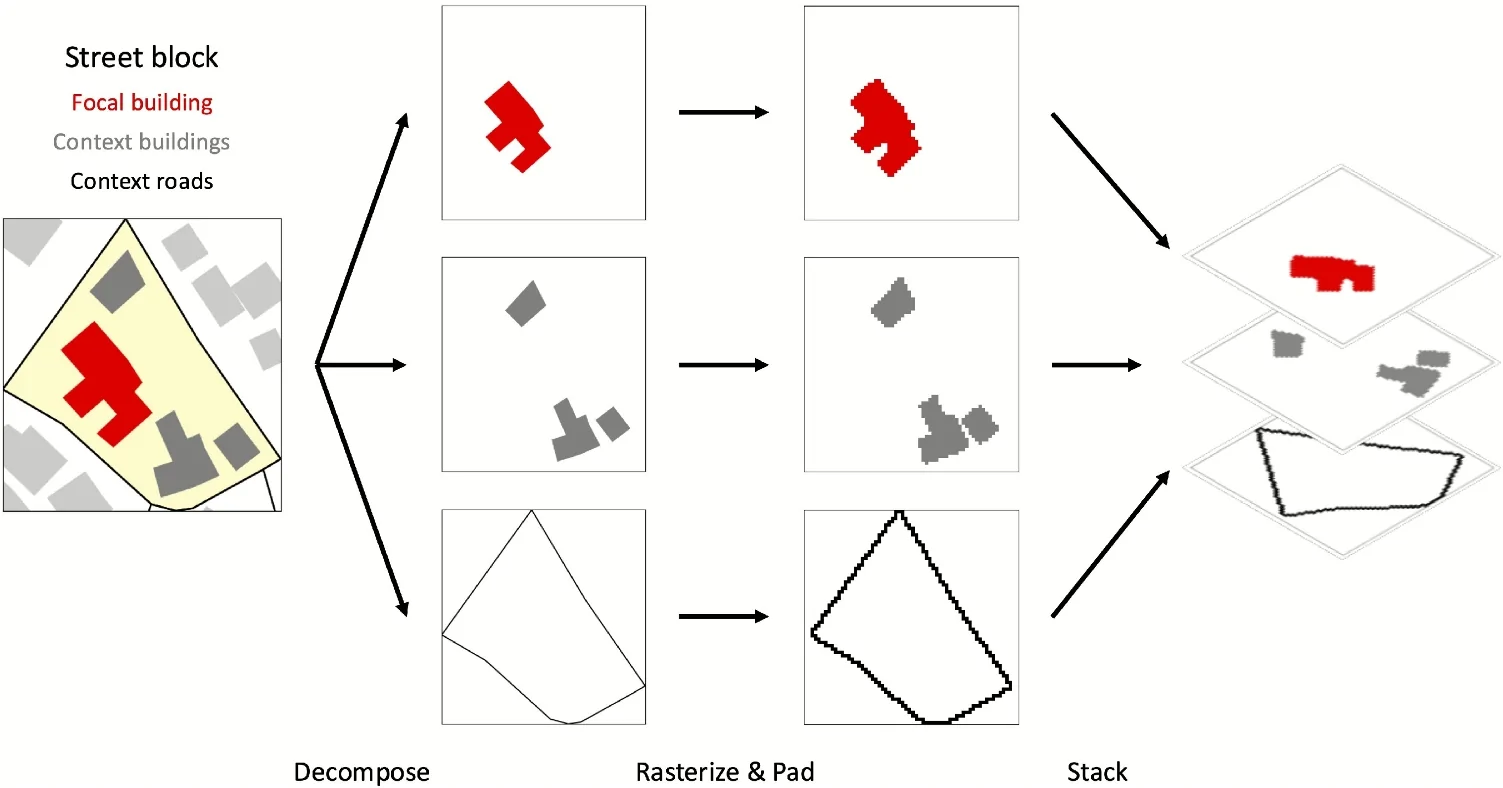
Graphical workflow of the proposed three-channel raster data model and its construction

Following the layer representation practices used in Courtial et al. (2022) and Fu et al. (2024), a given map of a street block, containing a group of buildings bounded by the road network, is encoded into three channels. As illustrated in Fig. 4 the three channels store the focal building to be generalized, the context buildings, and the context roads, respectively. In practice, the first step is to extract and separate the vector geometries of these map objects. The bounding box (BBOX) of the focal building’s street block is subsequently used to calculate the pixel resolution necessary for the street block to fit into a raster with a side length of 256 pixels according to Eq. 1.

1 $$\begin{aligned} \text {resolution} = \frac{\max (\text {BBOX width}, \text {BBOX height})}{256}. \end{aligned}$$

Next, the three separate map layers are rasterized using the computed resolution to generate three binary rasters that indicate the presence or absence of the focal building, context buildings, and context roads, respectively, at a given pixel. The created rasters are subsequently padded in the direction of the shorter side of the street block BBOX until they reach a common target shape of 256 x 256 pixels. The extent of all computed rasters corresponds to the extent of the street block. The raster shape of 256 x 256 pixels is empirically chosen given the map scale according to the suggestions provided by Touya et al. (2019), as it offers a good compromise between the sharpness of building boundaries and computational cost. Lastly, the three computed rasters are stacked together according to the proposed three-channel raster data model.

#### Heterogeneous Graph Data Model

In a vector map, the most important spatial information for contextual building generalization operator inference is the spatial relationships between map objects. Considering the different roles of a focal building, context buildings, and context roads within a street block, a heterogeneous graph data model is proposed. From a conceptual perspective, this is analogous to the outlined three-channel raster data model, as such models allow for the representation of multiple types of nodes and edges (Zhang et al. 2019) and therefore can capture the relationships between buildings and roads within a street block.

**Fig. 5:**
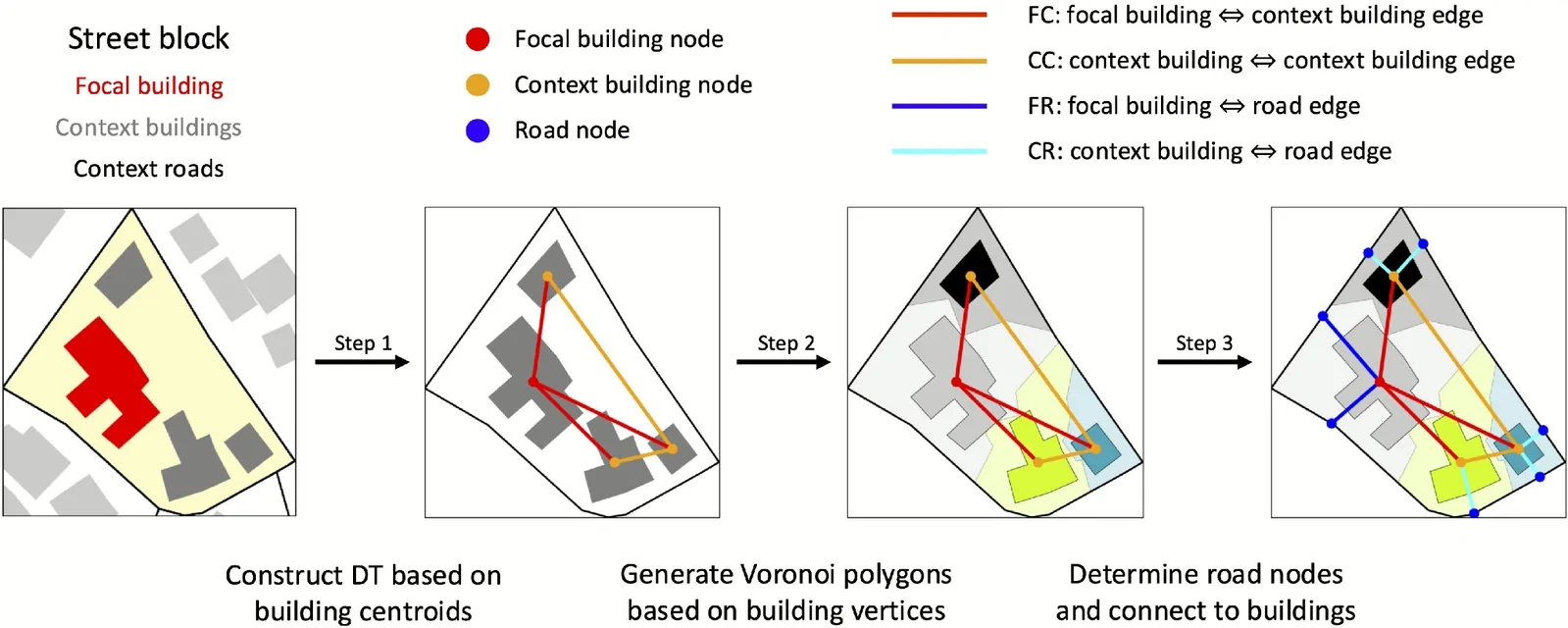
Graphical workflow of the proposed heterogeneous graph data model and its construction

As illustrated in Fig. 5, the proposed heterogeneous graph is composed of three types of nodes and four types of edges. Given the map of a street block, the focal building, context building, and road node are assigned to three types of nodes, such that they can be distinguished similarly to the proposed three-channel raster data model. The nodes representing the focal and context buildings are both geometrically extracted from the centroids of the buildings. Then, the Delaunay triangulation (DT) is constructed using the building centroids as seeds, and a proximity graph can be derived from the DT network, similar to the approach in Yan et al. (2019). As a consequence, two types of edges (FC: focal building *↔* context building; CC: context building *↔* context building) are constructed from the proximity graph of the buildings in the street block and added to the heterogeneous graph.

To represent the spatial relationships between buildings and roads in a street block, some vertices of the polyline geometries of context roads are sampled. As shown in Step 2 of Fig. 5, the Voronoi polygon based on its polygon vertices is generated, representing its region of influence on the other buildings within the street block (Yan et al. 2019). If the Voronoi polygon generated from the building touches the road network, the intersection between the Voronoi polygon and the road network determines the road segment associated with the respective building. Road nodes are determined by computing the intersection points between the road segment and the major and minor axes of the minimum bounding rectangle (MBR) of the building polygon. Afterward, in Step 3 of Fig. 5, the road nodes are subsequently connected to the building by an edge, ensuring that the orientation of the building is implicitly preserved in the graph structure. Road nodes are not connected to each other, as information associated with roads is only expected to be propagated to buildings during the learning process, rather than among the roads themselves. Such a process leads to two additional types of edges in the heterogeneous graph: FR (focal building *↔* road) edge and CR (context building *↔* road) edge.

Based on the built heterogeneous graph, 10 features are used to characterize different aspects of individual nodes and their contextual relationships, which are commonly used in automated map generalization. As shown in Table 1, the building nodes are described by five properties, including *position*, *size*, *shape*, *density*, and *orientation*, and each property is described by two features. Apart from *position*, the building node features cannot be easily transferred to road segments; therefore only *position* is chosen to characterize the road nodes. Additionally, the features from the *distance* property are used as edge weights for all edges in the graph. For more details on the heterogeneous graph data model, see (ANONYMIZED_1).^2^

**Table 1:** Building node features of the heterogeneous graph for a street block

Property	Feature	Calculation	Source
Position	Centroid x-coordinate	$\frac{1}{N} \sum _{i=1}^{N} x_i$	(Knura 2021)
	Centroid y-coordinate	$\frac{1}{N} \sum _{i=1}^{N} y_i$	
Size	Building area	$A_{\text {building}}$	(Yan et al. 2019)
	Building perimeter	$P_{\text {building}}$	
Shape	Convexity	$\frac{A_{\text {building}}}{A_{\text {CH}}}$	(Basaraner and Cetinkaya 2017)
	Equivalent rectangular index	$\sqrt{\frac{A_{\text {building}}}{A_{\text {MBR}}}} \cdot \frac{P_{\text {MBR}}}{P_{\text {building}}}$	
Density	Area of Voronoi polygon	$\frac{A_{\text {Voronoi}}}{A_{\text {block}}}$	(Xiao et al. 2024a)
	Impact area ratio	$\frac{A_{\text {building}}}{A_{\text {Voronoi}}}$	(Zhang et al. 2008)
Orientation	Orientation of MBR	/	(Duchêne et al. 2003)
	Wall average	/	(Hangouët 1998)

### Multi-modal Map Fusion

Given the great potential of multi-modal deep learning in significantly improving the performance of a wide range of vision and natural language recognition tasks (Huang et al. 2020; Mroueh et al. 2015), this study introduces a multi-modal learning paradigm to use the complementary information provided by the raster and vector map data modalities to make more informed contextual operator inference to generalize a focal building. The key to multi-modal learning is to fuse the information of each data modality. In this study, we implemented it based on a late fusion approach, which involves the integration of the outputs of separate models previously trained on individual modalities to construct an ensemble model that is capable of processing both modalities concurrently (Gadzicki et al. 2020). As illustrated in Fig. 3, the extracted raster features and graph features are directly concatenated into a fused feature. The following sub-sections detail the process used to extract features of each map data modality.

#### Raster Feature Extraction

**Fig. 6:**
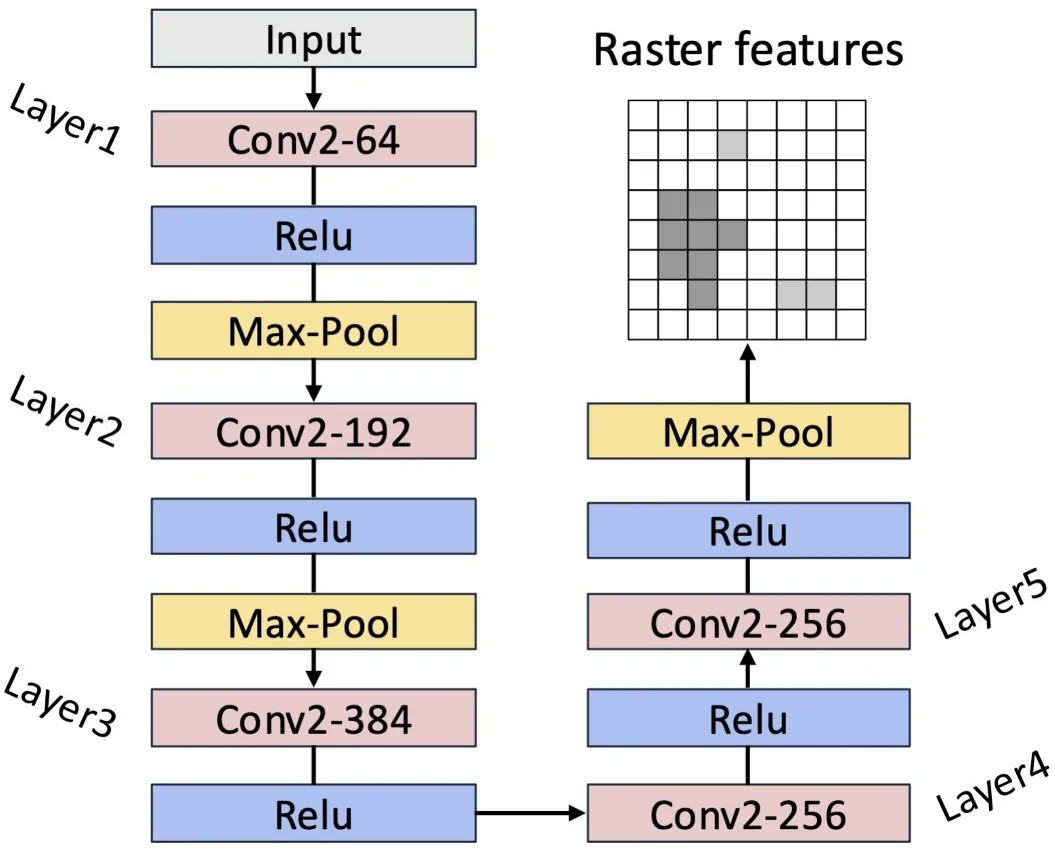
The AlexNet CNN architecture used for raster feature extraction

In our method, we adopted a classical and significant CNN architecture, AlexNet (Krizhevsky et al. 2017), as a backbone to extract raster map features from the three-channel raster data model. The rationale for using AlexNet is that the network has gained prominence for its exceptional performance in various conventional image classification tasks, while a prior study (Winkler 2023) has empirically demonstrated that a more sophisticated network does not necessarily improve the performance of raster map generalization. As shown in Fig. 6, the adopted CNN architecture is composed of five layers. Each of the first two layers and the last layer contains a convolutional module, an activation module, and a pooling module, while the middle Layers 3 and 4 do not include the pooling module to preserve potentially valuable raster map features.

#### Graph Feature Extraction

**Fig. 7:**
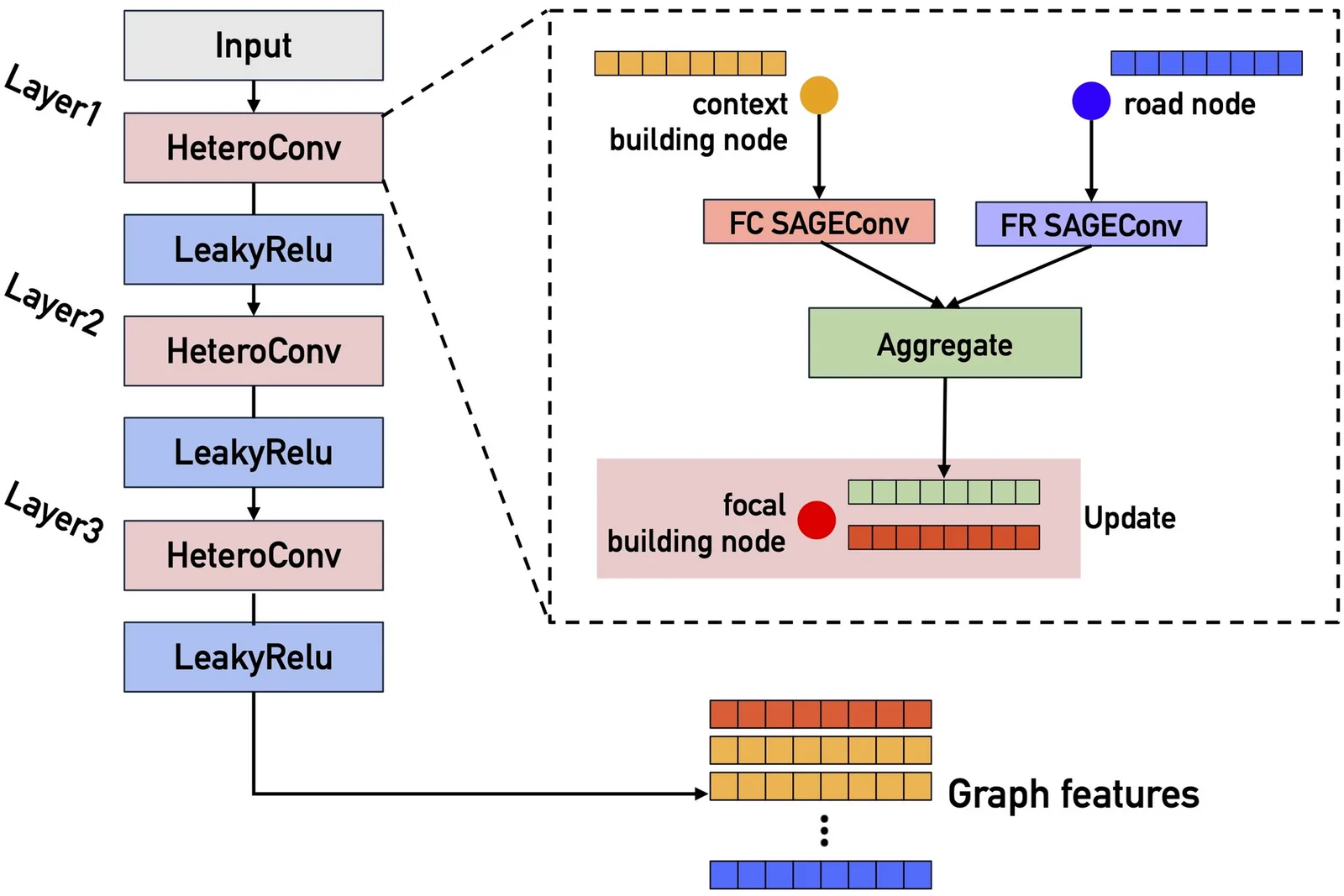
The three-layer HGNN architecture used for graph feature extraction

Given a heterogeneous street block graph, it is natural to learn representations that respect the semantic meaning of each node and edge type through heterogeneous graph neural networks (HGNNs) (Gong et al. 2024; He et al. 2024; Wang et al. 2022). In this study, as shown in Fig. 7, an HGNN composed of three heterogeneous graph convolutional (HeteroConv) layers (Zhang et al. 2019) is employed to extract the features of nodes contained in the heterogeneous street block graph. In each HeteroConv layer, for each node, a certain number of graph convolution modules are defined based on the types of edges connected to it. Then, the features from its neighboring nodes will be passed through these edge-specific graph convolution modules and aggregated to update the node’s features. As shown in the dashed-line box of Fig. 7, given a focal building node connecting a context building node and a road node, the features of the context building node will be processed through an FC SAGEConv module (Zhou et al. 2023), while the features of the road node will be processed through another graph convolutional module, FR SAGEConv. Afterward, the processed node features of the two types will be aggregated to update the focal building node features. Notably, we used the mean aggregation method for this purpose.

### MLP Classification Heads

After removing the classification heads, both the raster CNN and graph HGNN produce fixed-length feature embeddings for each sample: the CNN output is flattened into a one-dimensional vector, and the HGNN output corresponds to the embedding of the target node type. As shown in Fig. 3, the extracted raster features and the graph features are concatenated along the feature dimension, forming a joint representation whose size equals the sum of the raster and graph feature dimensions. This concatenated vector is then passed to four MLP classification heads, each of which will predict whether a specific map generalization operator should be applied to a focal building or not, by integrating complementary information from both modalities. For each classification head, the MLP is a three-layer structure consisting of a fully connected input layer, a ReLU hidden layer, and a fully connected output layer. The optimization of each MLP classification head’s parameters is based on the binary cross-entropy (BCE) loss. Given a set of *n* true binary labels $Y = \{y_1, \dots , y_n\}$, where $y_i \in \{0, 1\}$, and its associated set of predicted probabilities $\hat{Y} = \{\hat{y}_1, \dots , \hat{y}_n\}$, where $\hat{y}_i = [0, 1]$, the BCE loss $L_{\textit{BCE}}$ is calculated as shown in Eq. 2.

2 $$\begin{aligned} L_{\textit{BCE}} = -\frac{1}{n} \sum _{i=1}^{n} y_i \cdot \log (\hat{y}_i) + (1 - y_i) \cdot \log (1 - \hat{y}_i). \end{aligned}$$

## Experiment and Evaluation

We evaluated the performance of the proposed multi-modal learning methodology for contextual operator inference in building generalization by experimenting with multi-scale national map production data, maintained by the Swiss Federal Office of Topography (swisstopo). The experiments mainly aim to answer the following three questions:

1. To what extent can the multi-modal learning methodology improve the multi-label contextual generalization operator inference compared with the uni-modal models, that is, raster-based or graph-based? (Section Global Evaluation)
2. How will the operator inference performance of the proposed method vary with particular spatial characteristics, that is, the area (i.e., size) of street blocks and their urban vs. rural status? (Section Stratified Evaluation)
3. How will the road network, which is a critical context of buildings, influence the operator inference performance of the proposed method in building generalization? (Section Influence of Road Network)

### Experimental Setup

#### Data

**Fig. 8:**
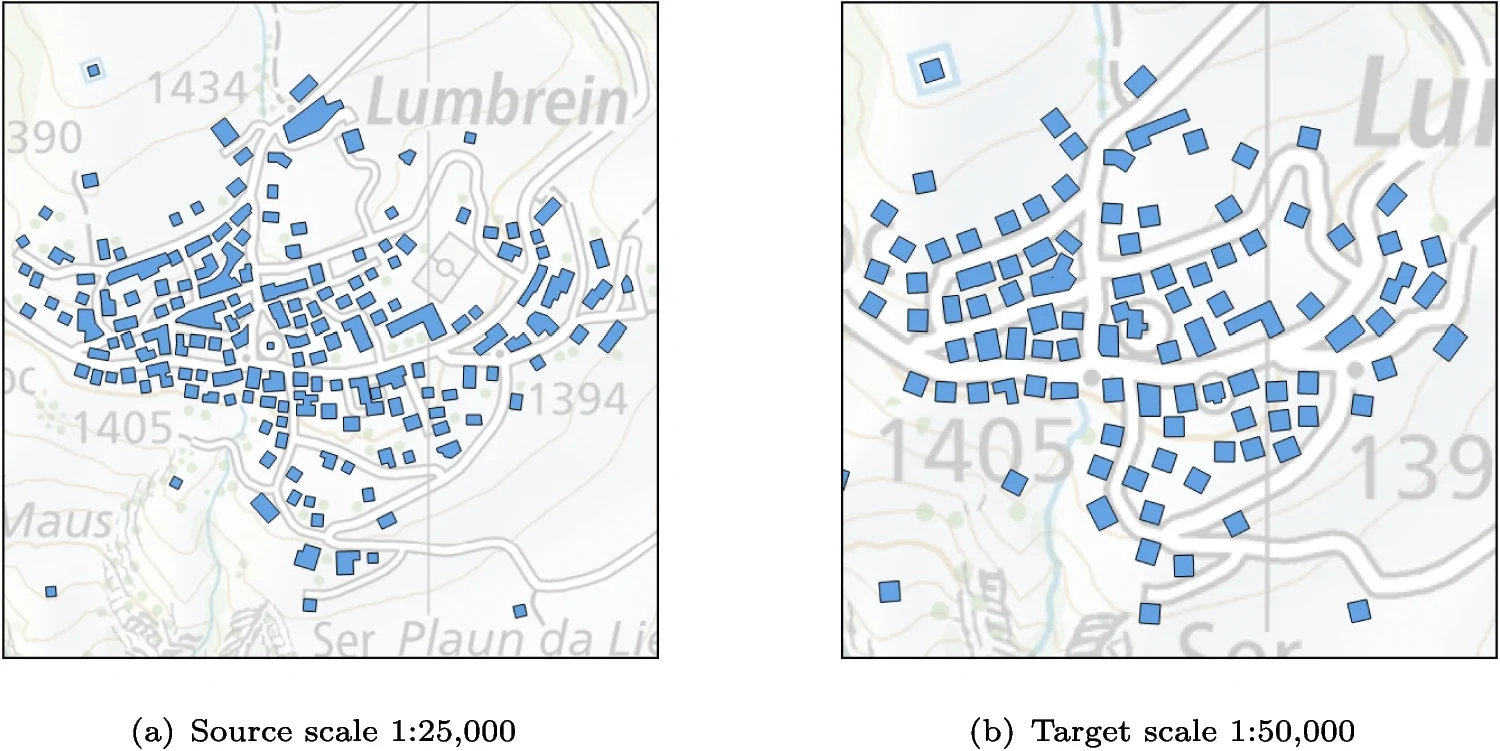
Buildings generalized to the investigated source and target scales (map data © swisstopo)

The building geometries used in this study originate from the DKM25 and DKM50 products of swisstopo (DKM = *digitales kartografisches Modell* in German, or digital cartographic model), containing vector geometries of all buildings across Switzerland, generalized to 1:25,000 and 1:50,000, respectively. Figure 8 illustrates the buildings at the two scales for a small map section. The scale transition from 1:25,000 to 1:50,000 was chosen since a mixture of contextual operators are predominantly applied in this scale range (Regnauld and McMaster 2007; Roth et al. 2011). By contrast, scale transitions above 1:25,000 are typically characterized by the application of operators displaying independent elements such as simplification or elimination, whereas transitions below 1:50,000 mainly involve typification above all other operators. While the DKM25 data are openly accessible under the product name Swiss Map Vector 25 (SMV25),^3^ the DKM50 data have not been publicly available and were made available to us by swisstopo upon request. The generalization process to produce these DKM datasets is conducted semi-automatically at swisstopo based on a commercial software system extended by bespoke cartographic tools configured to reach specific generalization objectives. As the tools of the semi-automated production process are applied under the control of expert cartographers, the results of this process are continuously validated visually and can thus be considered representing the approach and outcome of expert cartographers. Overall, the system achieves satisfactory generalization rates for roughly 80% of map features, necessitating manual edits for the remaining 20% (Duchêne et al. 2014). For the case of buildings, human intervention is particularly necessary in dense urban areas, where complex generalization situations may arise, as individual buildings are retained in the transition to DKM50.

Given the building geometries at the scales of 1:25,000 and 1:50,000, we partitioned the dataset into street blocks. All very large street blocks—mostly located in rural areas—containing more than 75 buildings at 1:25,000 were discarded, leaving 121,422 street blocks with 1,750,468 buildings. This corresponds to roughly 85% of the buildings in the Swiss National Maps at 1:25,000. For these retained buildings, the presence or absence of the four generalization operators in the scale transition from 1:25,000 to 1:50,000 was annotated using a semi-automated workflow, the details of which can be found in (ANONYMIZED_2). The outcome of this workflow was an initial version of a dataset to be used for training and validation of the proposed methodology for multi-modal contextual operator inference.

**Fig. 9:**
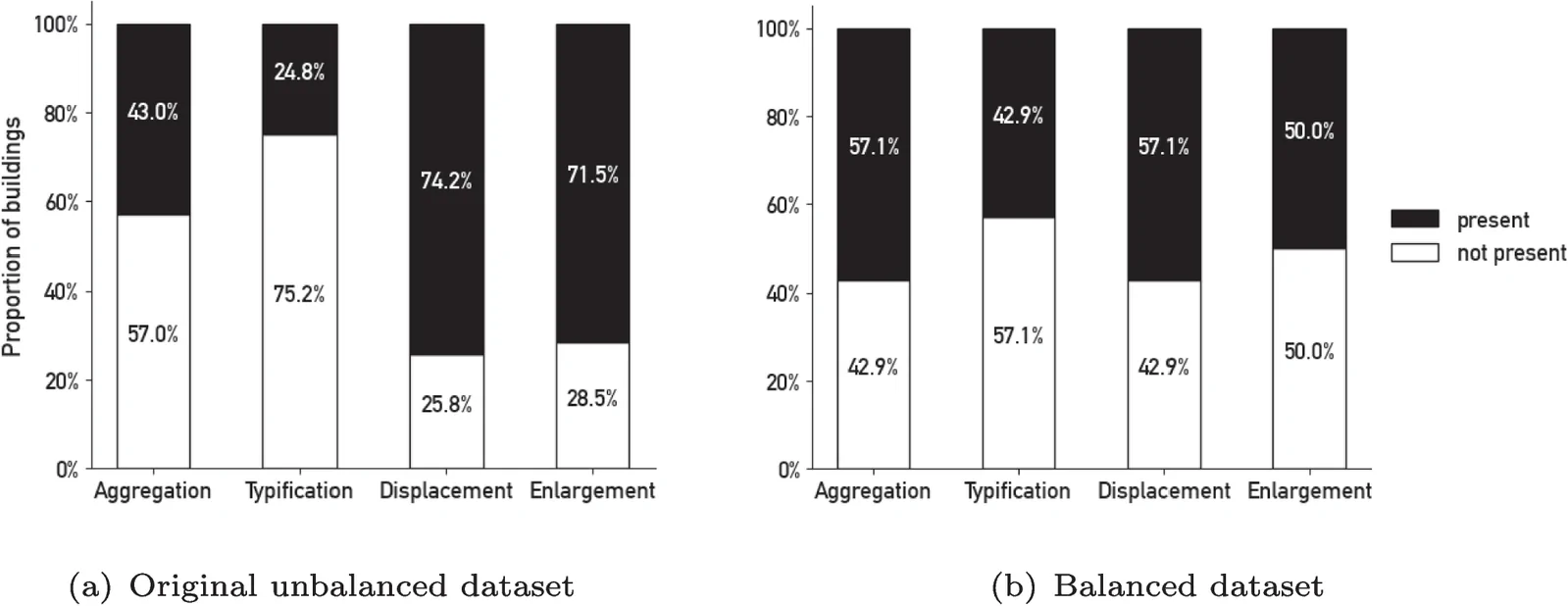
Frequency distribution of contextual generalization operators detected in the training dataset before and after data balancing

However, due to the high imbalance in the occurrence of the four individual generalization operators derived from the dataset (see Fig. 9(a)), we applied a label powerset (LP)-based hybrid resampling technique (Charte et al. 2013, 2015) to obtain a balanced dataset (see Fig. 9(b)). As a consequence, this resulted in a sample of 125,000 non-eliminated focal buildings, 80% of which were taken as the training set, while the remaining 20% formed the validation set. Furthermore, a separate, imbalanced test set was derived by randomly drawing a sample of 25,000 non-eliminated buildings from the original dataset that excludes the training and validation sets, which better reflects the model’s ability to classify the operators on a real map, where operators are highly imbalanced.

#### Implementation Details

**Fig. 10:**
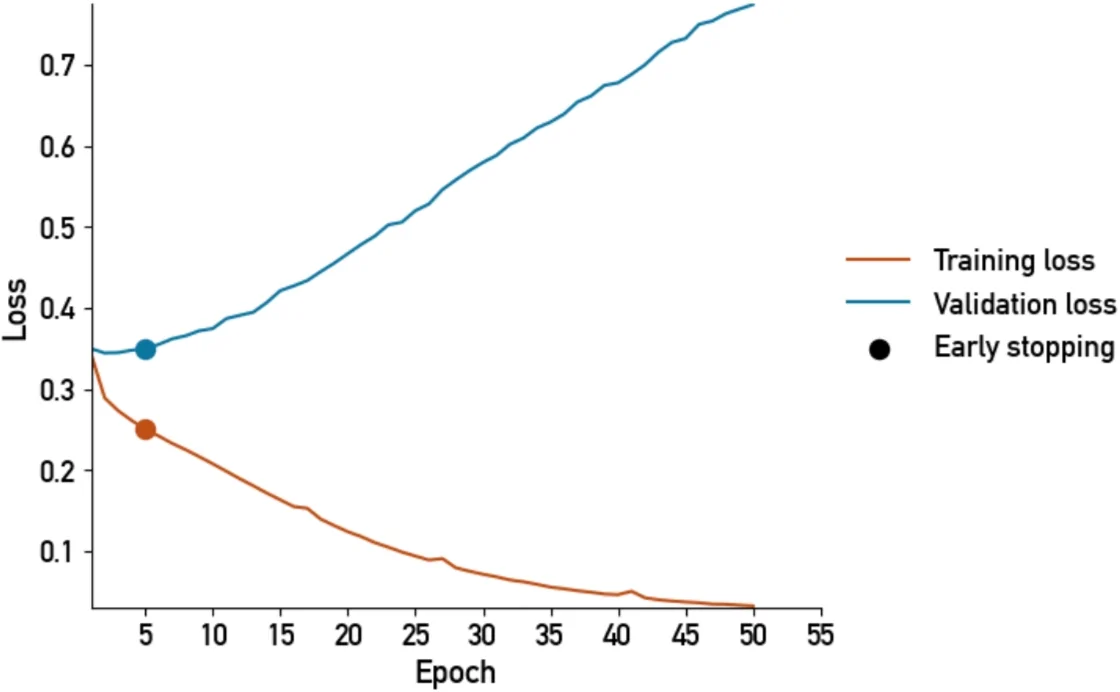
Loss curves for the multi-label, multi-modal model

The proposed multi-modal model was implemented in PyTorch and PyTorch Geometric (PyG). Given the large number of 100,000 training samples, a large batch size of 512 is chosen to train the models, which accelerates processing times. During training, the adaptive moment estimation (Adam; Kingma and Ba 2015) optimizer with a learning rate of $5 \cdot 10^{-4}$ was applied. Early stopping was used to determine the appropriate number of epochs for model training by identifying the epoch at which the model starts to exhibit overfitting for the validation dataset (Ying 2019). Figure 10 shows the progression of training and validation loss as the multi-modal model is trained over the course of the first 50 epochs. Judging from the validation loss curves, the model starts to display excessive overfitting after just 5 epochs, which is therefore chosen as the number of epochs for early stopping.

To compare our proposed multi-label, multi-modal learning method with the solely raster-based or graph learning method, we trained three models: a pure CNN model with the raster modality as shown in Fig. 6, a pure HGNN model with the graph modality as shown in Fig. 7, and our proposed multi-modal model fusing both the raster and graph modalities.

#### Evaluation Metrics

We adopted the commonly used classification evaluation metrics, including *precision*, *recall*, and *F1 score*, to evaluate the performance of the models for multi-label contextual operator inference in building generalization. In the calculation of the metrics, the presence of an operator denotes a positive case, while the opposite, that is, the absence of an operator, is regarded as a negative case. However, instead of using the commonly used 0.5 as a threshold to determine the presence/absence, the receiver operating characteristic (ROC) and precision-recall (PR) curves were generated for the trained models based on the validation set to identify the optimal separation threshold for each operator. The final threshold used to evaluate the performance of the models corresponds to the arithmetic mean between the optimal thresholds determined through both curves. As a result, the thresholds to evaluate the trained models are shown in Table 2.

**Table 2:** Threshold setting to evaluate the operator inference performance for the three trained models

Operator	CNN	HGNN	Ours (CNN + HGNN)
Enlargement	0.50	0.40	0.50
Aggregation	0.55	0.45	0.50
Typification	0.45	0.40	0.40
Displacement	0.45	0.55	0.50

### Global Evaluation

Global evaluation refers to the justification of operators inferred by our proposed multi-modal learning model and its comparison with the other two uni-modal learning methods, i.e., CNN and HGNN. The evaluation initially focuses on the presence of individual operators for a focal building (Section Individual Operators) and then on the presence of a set of operator combinations applied to the same focal building (Section Operator Combinations).

#### Individual Operators

The inference performance of four building generalization operators is reported in Table 3. For the *enlargement* operator, the performance achieved by our proposed multi-modal model is the same as that by the HGNN model, with a precision of 0.98, a recall of 0.88, and a F1 score of 0.93. In contrast, the CNN model had slightly lower evaluation metrics (precision: 0.97, recall: 0.86, and F1 score: 0.91). For the *aggregation* operator, our model achieved better precision (0.01 higher than the second-best CNN model) and F1 score (0.01 higher than the second-best CNN model), while the CNN model achieved the highest recall with 0.87, 0.01 higher than our model. For the *typification* operator, the precision of our model is 0.52, equal to that of HGNN and 0.01 higher than that of CNN. Although the CNN model had the highest recall with 0.69, our model resulted in the highest F1 score, 0.01 higher than the second-best CNN model. Lastly, for the *displacement* operator, the CNN model performed best across all three metrics, although the precision was the same as the other two models and the F1 score was equal to that of our proposed multi-modal model.

**Table 3:** Operator inference performance of the trained three models using different map data modalities. Numbers are given as mean values of the evaluation metrics, and bold numbers denote the highest value across the three models

Operator	Metric	Model (Modality)		
		CNN (Raster)	HGNN (Graph)	Ours (Raster + Graph)
Enlargement	Precision	0.97	**0.98**	**0.98**
	Recall	0.86	**0.88**	**0.88**
	F1	0.91	**0.93**	**0.93**
Aggregation	Precision	0.73	0.67	**0.74**
	Recall	**0.87**	0.84	0.86
	F1	0.79	0.75	**0.80**
Typification	Precision	0.51	**0.52**	**0.52**
	Recall	**0.69**	0.64	0.68
	F1	0.58	0.57	**0.59**
Displacement	Precision	**0.95**	**0.95**	**0.95**
	Recall	**0.73**	0.56	0.72
	F1	**0.82**	0.71	**0.82**

#### Operator Combinations

Since the generalization of a building frequently involves more than one operator, which imposes a higher level of difficulty on the operator inference of a DL model, we evaluated the actual operator combinations for each focal building in the test set. As shown in Table 4, for *none* or a *single* operator set, our proposed model had a higher F1 score than the other two models in inferring the *none* generalization and *displacement*, 0.01 higher than the second-best CNN model, respectively. However, the best performance for the *enlargement* was achieved by the CNN model, with an F1 score of 0.45, followed by our model with an F1 score of 0.43. In terms of the set of two-operator combinations, that is, the combinations of *displacement* and *enlargement*, and *aggregation* and *displacement*, our multi-modal model got the highest F1 score, 0.48 and 0.13, respectively, while the second-best CNN had 0.45 and 0.11, respectively. Furthermore, for the set of three-operator combinations, including the combinations of *aggregation*, *displacement*, *enlargement*, and *aggregation*, *typification*, *displacement*, the best performance of the former set went to our model, with an F1 score of 0.29, and the HGNN resulted in a higher F1 score (0.22) than the other two models. Regarding the most complicated operator set involving all four operators, the CNN model and our model had the equivalent F1 score, 0.5, while the F1 score of the HGNN model was 0.04 lower.

**Table 4:** F1 scores for inference of operator combinations by the three trained models. The bold numbers refer to highest performance

Operator set	Model (Modality)		
	CNN (Raster)	HGNN (Graph)	Ours (Raster + Graph)
None (= no generalization applied)	0.22	0.21	**0.23**
Displacement	0.41	0.33	**0.42**
Enlargement	**0.45**	0.31	0.43
Displacement, Enlargement	0.45	0.22	**0.48**
Aggregation, Displacement	0.11	0.10	**0.13**
Aggregation, Displacement, Enlargement	0.25	0.23	**0.29**
Aggregation, Typification, Displacement	0.18	**0.22**	0.21
Aggregation, Typification, Displacement, Enlargement	**0.50**	0.46	**0.50**

### Stratified Evaluation

To investigate the impact of spatial configurations on the multi-operator inference for building generalization, which is prone to being neglected in the global evaluation, we conducted a stratified evaluation considering two spatial configurations: the area (i.e., size) of street blocks, and their urban vs. rural status. Focusing on the multi-modal model, we report the ROC AUC results to demonstrate our trained model’s inference and discrimination capacity for each operator.

#### Street Block Area

The evaluation stratified by street block area involves dividing the samples in the test set into four distinct groups, each representing a respective quartile range of the areas covered by street blocks. The stratified evaluation results are reported in Table 5, in which the first quartile contains the buildings belonging to the smallest 25% of street blocks, whereas the largest blocks 25% are contained in the fourth quartile. We can see a general trend of increasing ROC AUC values with increasing area quartile. Specifically, the ROC AUC of the *aggregation* operator increases from 0.82 in the first quartile to 0.88 in the fourth quartile, the *typification* operator from 0.71 to 0.83, the *displacement* operator from 0.67 to 0.77, and the *enlargement* operator from 0.92 to 0.96.

Although the ROC AUC values of the *inference* capacity of all four operators show consistently increasing trends with larger street block areas, the *discrimination* capacity of the trained multi-modal classification models remains at different levels across operators. The model performs best for the *enlargement* operator, with the ROC AUCs of the four quartiles all above 0.90, followed by the *aggregation* operator still at a good level as the ROC AUC values of the four quartiles are all above 0.80. Moreover, the performance for the *typification* inference varies between the fair level and good level, with ROC AUC values between 0.71 and 0.78 in the first three quartiles and 0.83 in the fourth quartile. The operator inferred most poorly, but still at a fair level, is *displacement*, with 0.67 in the first quartile and between 0.70 and 0.77 in the last three quartiles.

**Table 5:** ROC AUC upon stratification by street block area quartile

Operator	Area quartile			
	1st	2nd	3rd	4th
Enlargement	0.92	0.95	0.95	0.96
Aggregation	0.82	0.85	0.86	0.88
Typification	0.71	0.73	0.78	0.83
Displacement	0.67	0.70	0.70	0.77

#### Urban vs. Rural Status

Compared to rural areas, urban areas on topographic maps exhibit a higher density of map features. Therefore, urban areas usually require a higher degree of generalization with an increased use of complex contextual generalization operators, such as typification or displacement, to address cartographic conflicts arising from densely packed map objects (Mustière and Moulin 2002; Ruas and Mackaness 1997; Spiess et al. 2005). To evaluate the performance of the proposed model with respect to the urban vs. rural status of the street blocks, each block was assigned the status *urban* or *rural* based on its dominant land use class according to the Swiss Land Use Statistics (more details in (ANONYMIZED_1)). The evaluation results stratified by the urban vs. rural status are shown in Table 6. For buildings located in street blocks classified as urban, the ROC AUCs of the *enlargement*, *aggregation*, *typification*, and *displacement* inference are 0.94, 0.83, 0.72, and 0.71, respectively. In contrast, operator inference performance on building generalizations in rural street blocks show a consistently higher performance than their urban counterparts, with ROC AUCs of 0.95, 0.88, 0.82, and 0.76 for the four individual operators, respectively.

**Table 6:** ROC AUC upon stratification by urban vs. rural status

Urban vs. rural status	Operator			
	Enlargement	Aggregation	Typification	Displacement
Urban	0.94	0.83	0.72	0.71
Rural	0.95	0.88	0.82	0.76

### Influence of Road Network

To determine whether supplying the models (CNN, HGNN, multi-modal) with more contextual cartographic information in the form of the road network enclosing the street blocks is, in fact, beneficial for the operator classification task, the models were retrained after excluding the information associated with the roads from the training samples. The corresponding results are shown in Table 7. For the CNN model, the removal of the road-related features led to a 0.01 improvement of ROC AUC in the *typification* inference but a 0.07 drop in the *displacement* inference, while the ROC AUC in the *enlargement* and *aggregation* inference remained the same. For the HGNN model, the inference ROC AUC of the *aggregation* and *typification* both decreased by 0.01, but the ROC AUCs of the other two operators did not change. For the proposed multi-modal model, the removal of the road-related features had no effect on the classification of three of the operators, but caused a significant decrease in ROC AUC for the inference of *displacement*.

**Table 7:** Change in ROC AUC induced by removal of road-related features. Negative change implies worse classification performance when these features are excluded from model training

Model (Modality)	Operator			
	Enlargement	Aggregation	Typification	Displacement
CNN (Raster)	0.00	0.00	0.01	−0.07
HGNN (Graph)	0.00	−0.01	−0.01	0.00
Ours (Raster + Graph)	0.00	0.00	0.00	−0.06

## Discussion

### Advantages of Multi-modal Learning

**Fig. 11:**
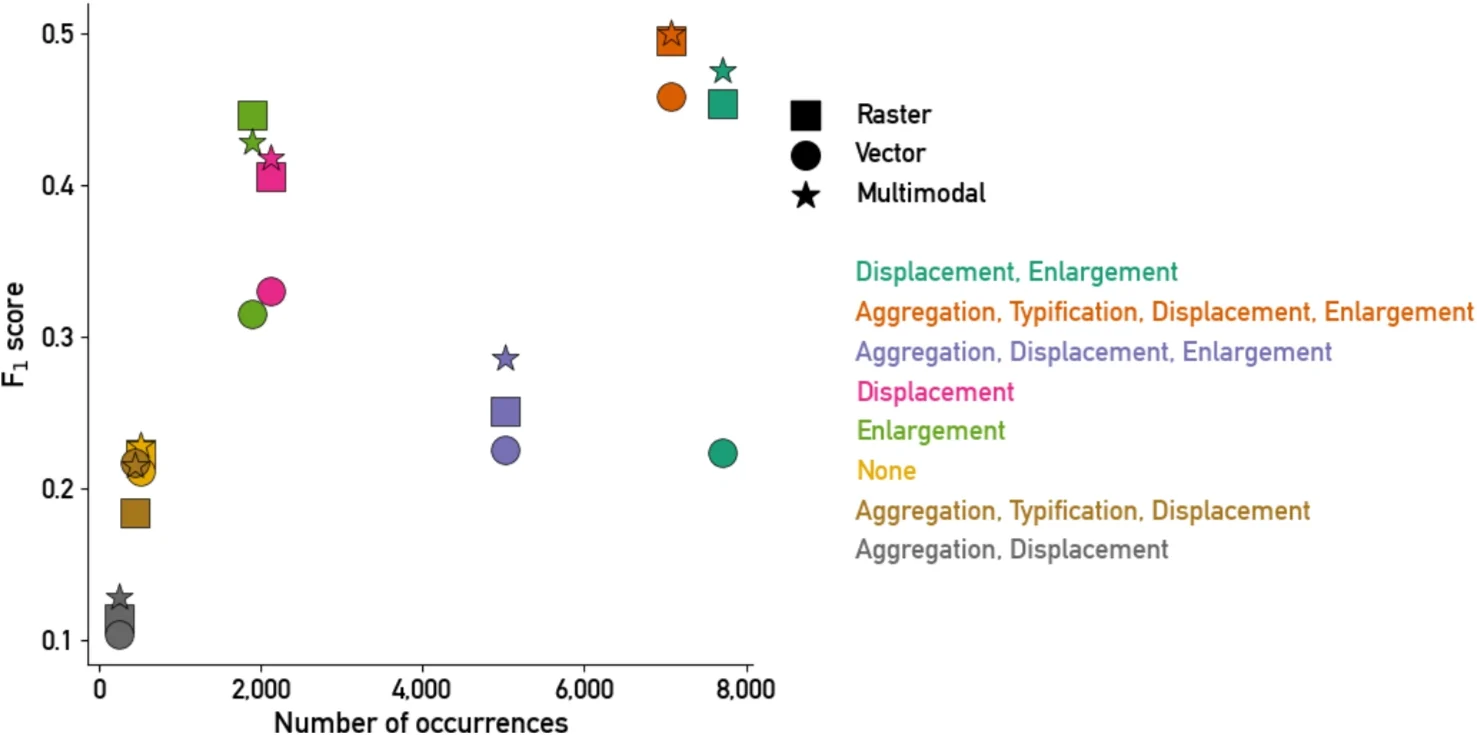
Relationship between operator combination prevalence and F$_1$ score

The global evaluation of individual operators, as elaborated in Section Global Evaluation, demonstrates that all three modalities exhibit similar capabilities for the inference of the four individual operators involved in building generalization, although our proposed multi-modal model achieved slightly higher performance in more metrics compared with the CNN and HGNN models. However, synthesizing the quantitative evaluations on more realistic generalization scenarios with operator sets in Section Operator Combinations and their correspondence with the number of occurrences as mapped in Fig. 11, the advantages of multi-modal learning become clearer. Specifically, the multi-modal model outperforms the other two models for the most frequent operator combination of *enlargement* and *displacement* (the dark green group in Fig. 11), and the third most frequent set containing *enlargement*, *aggregation*, and *displacement* (the purple group in Fig. 11). For the second most frequent set involving all four operators (in orange), as well as the other, less frequent sets, the proposed multi-modal model does not show an obvious advantage over the pure raster or the vector models—yet still reaches (close to) the top performance in most cases.

### Performance in Different Spatial Configurations

**Fig. 12:**
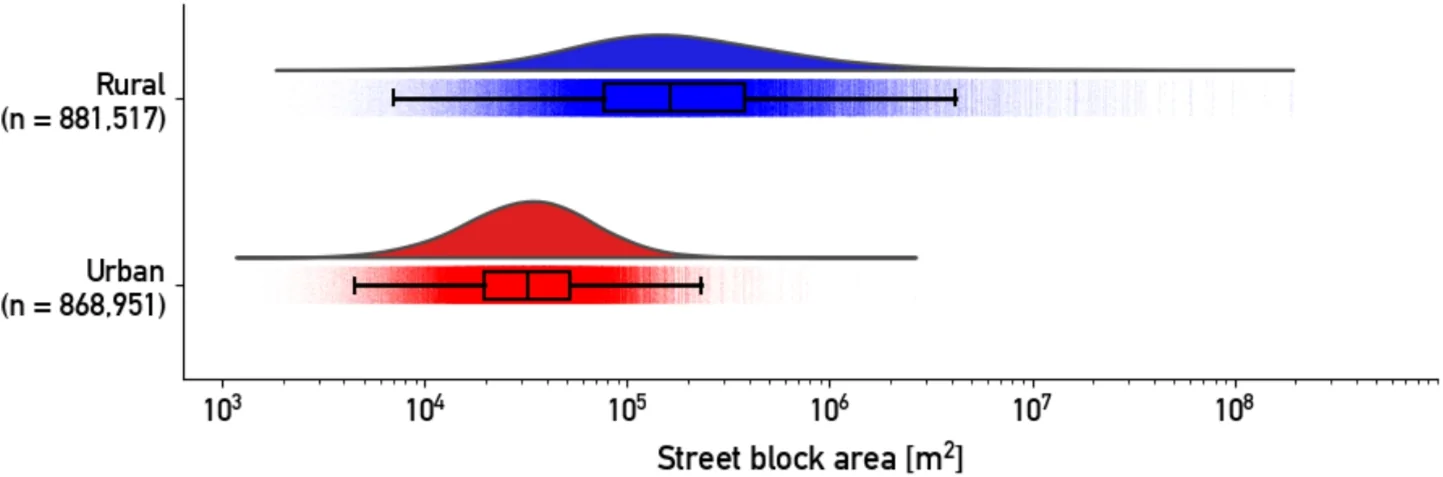
Street block area for buildings in urban and rural street blocks

Following the results of the stratified evaluation in Section Stratified Evaluation, all models exhibit an increase in performance with increasing street block area and on buildings in rural street blocks, except the raster model on enlargement. This finding is intuitive, since buildings in rural areas tend to be part of street blocks with much larger areas, as illustrated in Fig. 12. Furthermore, the findings can be considered representative since the training database evidently contains similar numbers of buildings in urban and rural contexts. In Section Evaluation Metrics, it was hypothesized that especially the raster data model may be susceptible to a scale effect, whereby performance would deteriorate with increasing area of street blocks due to the associated decrease in resolution. However, the results presented in Table 5 indicate that the opposite is true, as classification performance improves with increasing street block area.

Although there is no clear explanation for this inverse scale effect from a technical perspective, it can likely be attributed to insights derived from traditional map generalization practice. First, the higher performance observed for buildings within large street blocks can be linked to the fact that these blocks are predominantly situated in rural areas. Compared to urban contexts, the generalization of buildings in rural areas is generally considered an easier task, as the density of map objects is much lower and thus there is more room available to ‘maneuver’ and the operator combinations required tend to be less complex (Mustière and Moulin 2002; Ruas and Mackaness 1997; Spiess et al. 2005). Second, larger street blocks provide additional cartographic context that can facilitate the decision regarding operators. Transferring these insights to a DL context, it seems logical that the models are more adept at performing the generalization operator prediction for buildings located in rural areas.

### Influence of Road Network

**Fig. 13:**
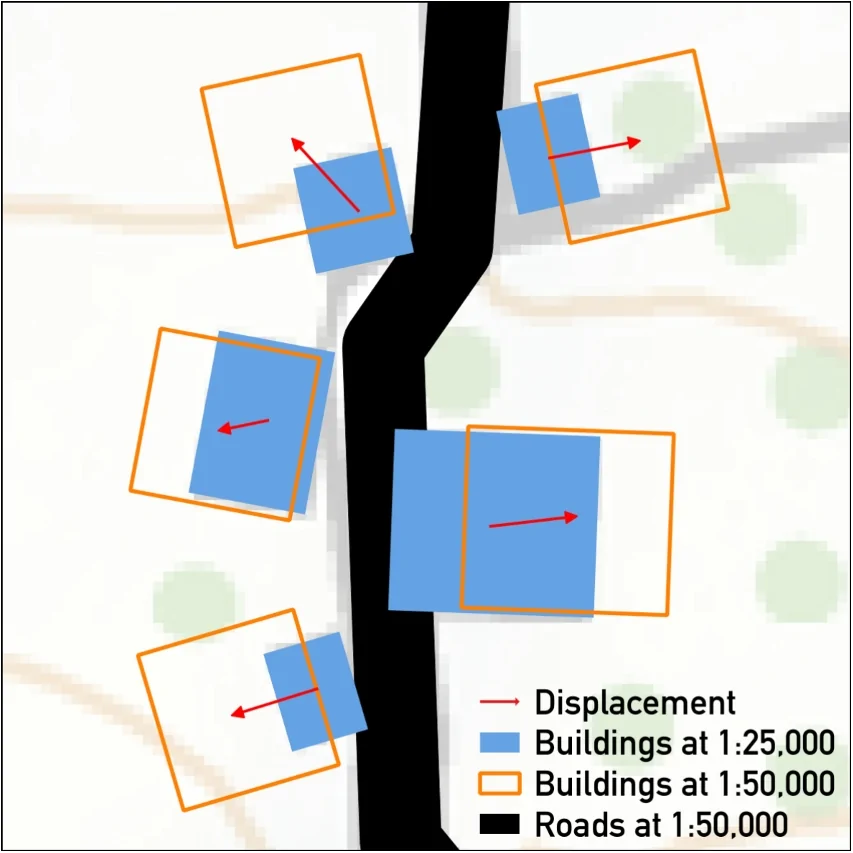
Application of the displacement operator necessitated by the generalization (i.e., enlargement of symbol width) of the roads (map data © swisstopo)

As elaborated in Section Influence of Road Network, for all three modalities, the ROC AUC values for *enlargement* and *aggregation* are not significantly affected by the removal of road-related features. This is consistent with expectations from traditional generalization practice, as the application of these two operators is not expected to be considerably influenced by the layout of the road network. For the raster and multi-modal models, the removal of road-related features evidently leads to a substantial decrease in ROC AUC for the *displacement* operator, implying better performance on this operator if the roads are included for training and prediction. Again, this observation is in line with traditional generalization practices, as the enlargement (i.e., widening) of road symbols when transitioning to smaller scales commonly necessitates the displacement of buildings situated along roads. This phenomenon is illustrated in Fig. 13, whereby the amount of available map space dictates the application of *displacement*. A similar effect can be observed for the vector-based model, which exhibits a slight decrease in performance for typification when roads are excluded (note: typification invariably also includes displacement). Again, this is intuitive, as the layout of the road network constrains the amount of available map space, which is critical for determining building density and therefore also the decision of whether typification should be applied or not (Lee 1996; Regnauld and McMaster 2007).

However, the fact that the performance of the graph model does not change for the enlargement and displacement operators when road-related features are excluded suggests that the developed graph construction method may not be the optimal solution to encode structural knowledge associated with roads. Nonetheless, in general, the results obtained demonstrate that including additional cartographic context in the form of the road network during the training process of the different DL models has the potential to increase the quality of building generalization operator inference. Importantly, the findings further suggest that DL models can learn cartographic knowledge that is consistent with traditional generalization practices.

### Visual Investigation

**Fig. 14:**
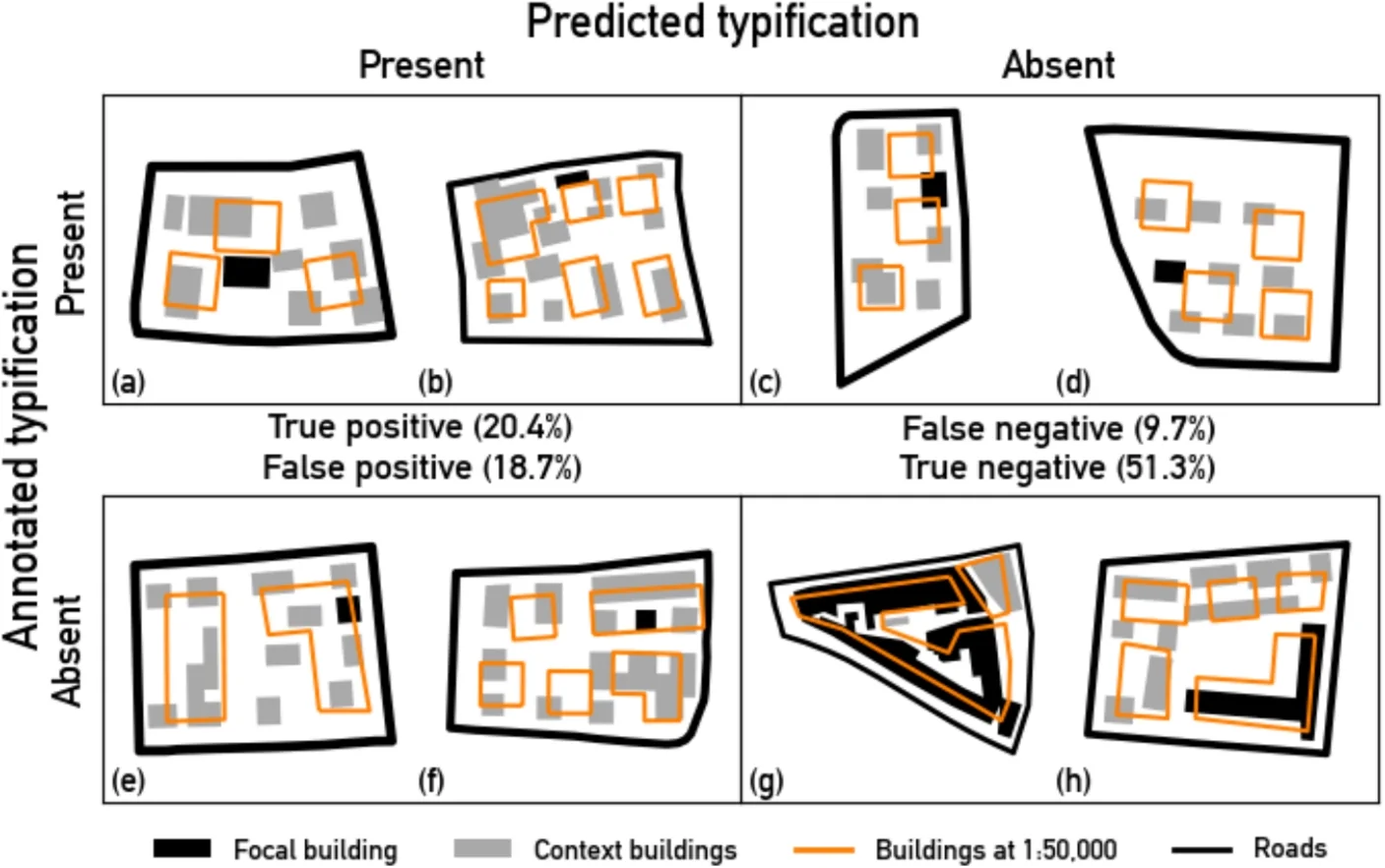
Visual evaluation of model performance for typification (map data © swisstopo)

Table 3 above showed that the worst performance is rendered when classifying typification, warranting further investigation. Figure 14 illustrates classification results produced by the multi-modal model in classifying typification. The examples are categorized into *true positive*, *true negative*, *false negative*, and *false positive* by comparing the predicted operator with the operator that was annotated in the test dataset. The corresponding illustrations for the remaining investigated operators can be found in Section Appendix A: Miss-inferred Examples of Contextual Operators.

Examples (a) and (b) demonstrate that the model correctly recognizes that the focal buildings in dense street blocks should be typified. On the other hand, examples (g) and (h) show that typification is appropriately classified as absent for prominent buildings with distinctive shapes in compact street blocks, since the preservation of these buildings is important for navigational purposes. However, examples (c) and (d) illustrate that the model fails to identify the presence of typification for small buildings in dense street blocks. Finally, examples (e) and (f) highlight that some aggregation operations are identified as typification, indicating that the model encounters difficulties in distinguishing between the two operators. Ultimately, the misclassifications can be largely attributed to the semi-automated operator annotation procedure used to generate the training dataset. During this process, it was often difficult to distinguish whether a building had undergone typification or had been subjected to the other operators, a distinction the model subsequently also struggles to replicate.

### Implications, Limitations, and Future Research

#### Implications

Overall, the study reported in this article has demonstrated how deep learning models can be used to infer contextual operators for building generalization and thus how DL can be used to learn and acquire cartographic knowledge. We consider this in itself a major contribution, along with the findings regarding the success of multi-operator learning, as well as the superior performance of the multi-modal model compared to the uni-modal models. In a real-world automated map generalization scenario, the model can assist in, or even free cartographers from, defining rules and parameters to decide building generalization operators on a case-by-case basis, which is typically followed by using specific algorithms for inferred operators to carry out the generalization process. This process can not only take full use of existing human-made multi-scale maps, but also improve the level of automation of map generalization with varying building contexts.

#### Limitations

Nevertheless, there is still room for improvement. The following assessment lists a range of limitations of this present work, none of which we consider a major impediment, however. First, the proposed approach is limited from a conceptual point of view, as it assumes that buildings are generalized individually rather than collectively. Therefore, it cannot account for cartographic conflicts and inconsistencies evoked by interference or knock-on effects between operators. From the perspective of the DL models, the experiments suggest that the multi-modal model does not show a monotonously decreasing trend in the training loss curve and is prone to overfitting (see Fig. 10), which may be mainly caused by the poor capacity in inferring the occurrence of the *typification* operator while the used BCE loss should sum over all types of contextual operators. Additionally, the imbalanced co-occurrence of contextual operators in the dataset may have imposed further difficulties on the training of the model, although the total volumes of the individual operators had been resampled and balanced. Also, the proposed approach does account for the potential interaction crossing raster and graph modalities, which may impede the upper bound of multi-modal learning (Chen et al. 2019; Wenderoth et al. 2025). Furthermore, the encoding procedure of street blocks as heterogeneous graphs conceived for the vector model is likely too complex to be efficient. Finally, the stratified sampling approach devised to address the imbalance present within the frequency distribution of the generalization operators displays limited effectiveness due to excessive oversampling.

#### Future Research

To address the identified limitations, future studies should more systematically seek to explore novel DL architectures (e.g., attention-based models, Manzoor et al. 2023; and foundation models, Lu et al. 2025; Zhao et al. 2025) that are capable of simultaneously predicting the presence or absence of individual operators for all buildings in a street block, while this study mainly aimed to identify a new learnable task by DL models for research in map generalization. Additionally, the remaining problems associated with operator imbalance call for the development of tailored resampling and data augmentation approaches, the exploration of alternative loss functions, and the implementation of ensemble methods. The encoding of additional procedural domain knowledge and the application of techniques from the domain of explainable AI have the potential to make meaningful contributions to knowledge acquisition and to the explainability of DL-based map generalization approaches (Fu et al. 2024). Finally, future research should strive to investigate alternative learning frameworks leveraging training datasets with fuzzy labels, such as pseudo-labeling learning (Zhao et al. 2023, 2025), and to develop sophisticated evaluation strategies that better capture the ambiguities intrinsic to the generalization process. All of these developments are paramount to truly ushering in DL as the next paradigm for end-to-end automated map generalization.

## Conclusions

This article focused on knowledge acquisition for map generalization using deep learning models, and as such opened a new line of research that was previously untouched in the literature. We formulated a new deep learning task, *multi-label contextual operator inference*, for process recognition and modeling in building generalization. Given the different advantages of raster and vector maps for generalization, we hypothesized that the integration of the two types of map data modalities could further benefit the *multi-label contextual operator inference* task. Hence, we proposed a multi-modal learning model to infer four contextual generalization operators commonly used on buildings, including *enlargement*, *aggregation*, *typification*, and *displacement*, after concatenating the raster and vector features extracted by an AlexNet model and an HGNN model. Our study resulted in the following findings:

- Overall, multi-modal learning outperforms uni-modal learning for generalization scenarios involving multiple operators, in particular the combination of *enlargement* and *displacement*.
- Although multi-modal learning does not show clear advantages over uni-modal learning in the evaluation of individual operator inference, all approaches achieved F1 scores greater than 0.8, except for the *typification* operator. This indicates the promising capability of DL approaches in acquiring procedural knowledge on building generalization.
- The stratified evaluation shows the higher performance in evaluation metrics when inferring the operators to be applied to buildings located within large street blocks in rural areas, indicating the varying generalization difficulty of street blocks of different spatial configurations.
- Street networks play an important role in the inference of the *displacement* operator but exert only minor influence on the other contextual operators.

## Figures and Tables

**Fig. 15:**
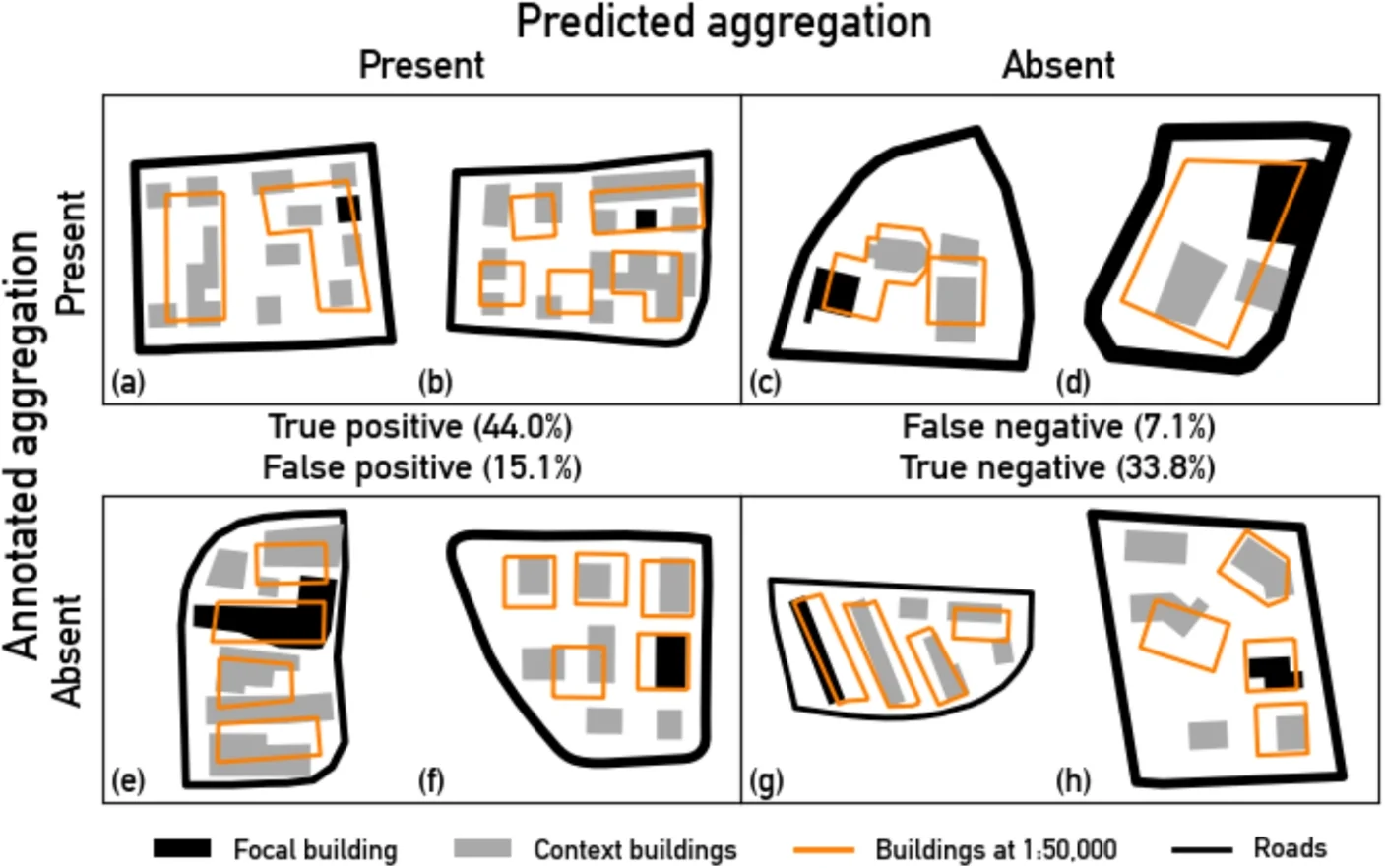
Visual evaluation of model performance for aggregation (map data © swisstopo)

**Fig. 16:**
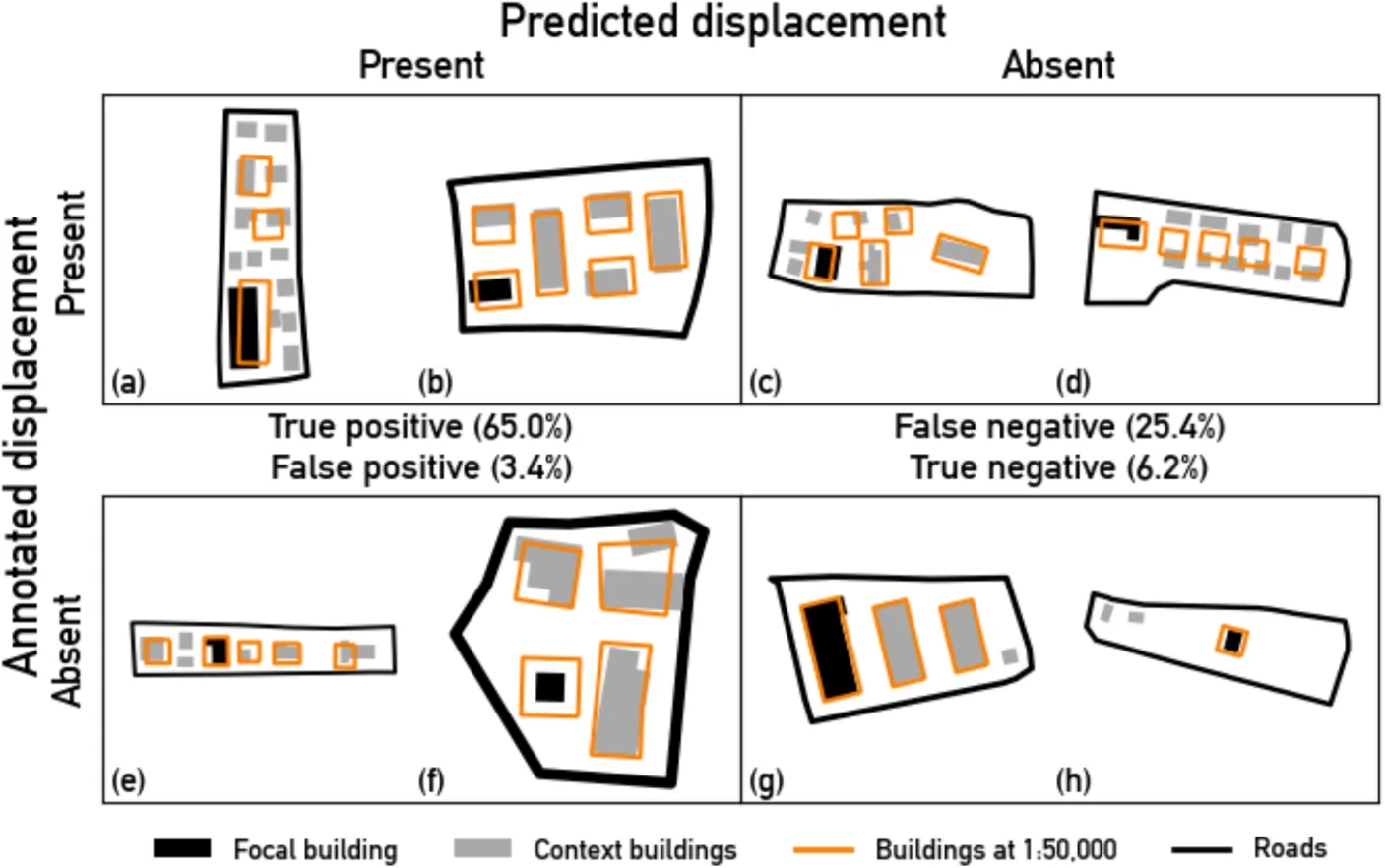
Visual evaluation of model performance for displacement (map data © swisstopo)

**Fig. 17:**
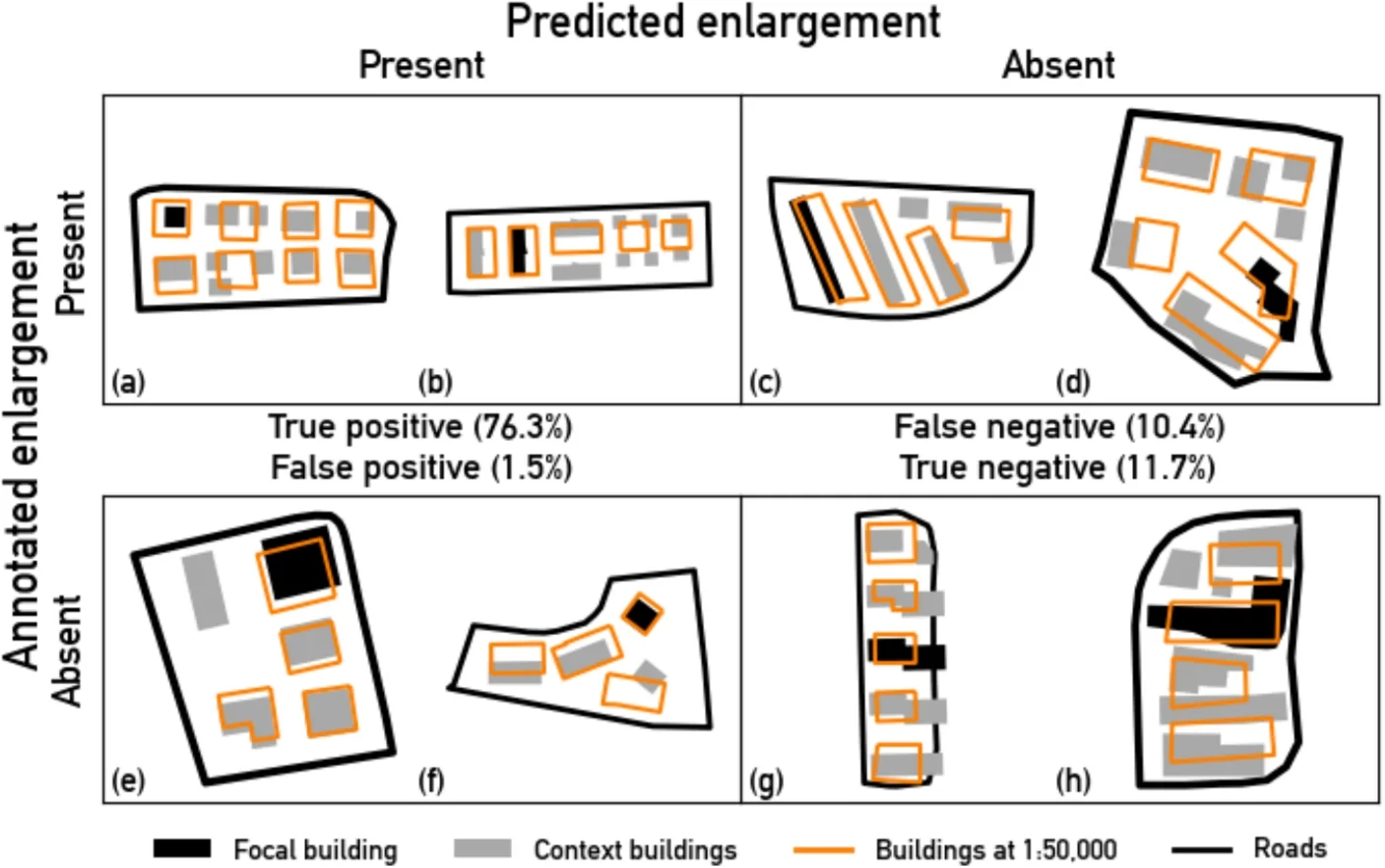
Visual evaluation of model performance for enlargement (map data © swisstopo)

## Data Availability

No datasets were generated or analysed during the current study.

## Appendix A: Miss-inferred Examples of Contextual Operators

### Aggregation

Figure 15 allows for a visual evaluation of the performance of the model in predicting the presence or absence of aggregation. Examples (a) and (b) show that the DL model is able to correctly identify that small focal buildings forming part of larger, dense groups are at risk of becoming indistinguishable to the human eye at the scale of 1:50,000, for which the application of aggregation is proposed (note that these were the cases that led to false positives in the prediction of the typification operator). Judging from examples (g) and (h), the model is further able to correctly recognize that for focal buildings with access to abundant map space aggregation is not necessary and other operators, such as displacement and enlargement, should be applied instead.

Examples (c) and (d) illustrate two cases in which aggregation was present in the underlying dataset, whereas the model predicted alternative operators. In both cases, sufficient map space is available, implying that the application of other operators, such as displacement or enlargement, could also provide satisfactory solutions, leading to confusion in the prediction. Finally, examples (e) and (f) illustrate instances where focal buildings are embedded within densely packed street blocks for which the model recommends aggregation, presumably due to the high building density. However, in the annotated map, conflicts are resolved by applying the typification operator, which is easily confused with the aggregation operator.

### Displacement

Figure 16 provides a visual assessment of model performance in predicting the presence or absence of displacement. Examples (a) and (b) illustrate that the model correctly recognizes that focal buildings along roads should be displaced away from the road network, because the road network is subject to significant enlargement when transitioning to 1:50,000. Furthermore, examples (g) and (h) demonstrate that the model appropriately classifies displacement as absent in cases where abundant space is available within the street block and thus displacement is evidently not necessary. On the other hand, example (c) shows an instance where the model proposes aggregation instead of displacement, and example (d) illustrates a case where the model fails to recognize the impending conflict of the focal building with the road network. In some instances, as exemplified in (e) and (f), the model struggles to recognize that there is sufficient map space available, predicting displacement in cases where it is not necessary. Again, these seem to be cases where typification and aggregation are at play within the street block.

### Enlargement

Figure 17 illustrates how the model performs in classifying enlargement for exemplary focal buildings. Examples (a) and (b) depict cases in which small buildings should be retained to conserve the structure of the street block. The model correctly recognizes that these buildings have to be enlarged in order to comply with the minimum dimensions stipulated by map specifications. Additionally, examples (g) and (h) demonstrate cases where the road network is significantly displaced to the point of intersecting the buildings, likely due to important map features in adjacent street blocks. In instances where the buildings risk extending beyond the street block, they should certainly not be enlarged, which the model correctly identifies. However, examples (c) and (d) show cases where the model fails to identify that narrow buildings should be enlarged. Finally, examples (e) and (f) depict instances where building enlargement is unnecessary, but the model predicts enlargement regardless.

## Footnotes

1 DKM25 and DKM50 are internal data products of swisstopo and thus not openly available. For DKM25 and DKM10, equivalent open data products are available for download from the swisstopo website under the product name Swiss Map Vector 25 (SMV25) and SMV10, respectively.

2 Note that anonymization of sources was carried out in accordance with the Submission Guidelines of this journal. The Editor has knowledge of these anonymized sources.

3 https://www.swisstopo.admin.ch/de/landeskarte-swiss-map-vector-25
